# Mechanobiological Modulation of *In Vitro* Astrocyte Reactivity Using Variable Gel Stiffness

**DOI:** 10.1021/acsbiomaterials.4c00229

**Published:** 2024-06-13

**Authors:** Julia C. Benincasa, Marianne I. Madias, Rebecca M. Kandell, Lina M. Delgado-Garcia, Adam J. Engler, Ester J. Kwon, Marimelia A. Porcionatto

**Affiliations:** † Department of Biochemistry, Escola Paulista de Medicina, Universidade Federal de São Paulo, São Paulo 04039032, Brazil; ‡ Department of Bioengineering, University of California San Diego, La Jolla, California 92093, United States

**Keywords:** glial scar, astrogliosis, matrix stiffness, astrocyte morphology, polyacrylamide gels

## Abstract

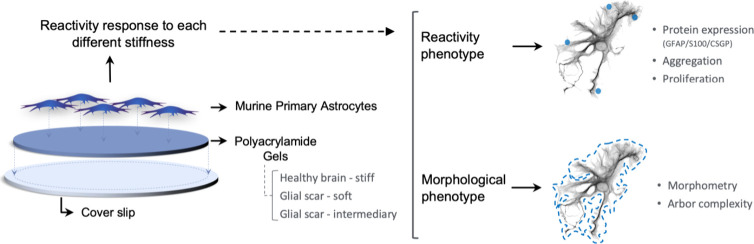

After traumatic brain injury, the brain extracellular matrix undergoes structural rearrangement due to changes in matrix composition, activation of proteases, and deposition of chondroitin sulfate proteoglycans by reactive astrocytes to produce the glial scar. These changes lead to a softening of the tissue, where the stiffness of the contusion “core” and peripheral “pericontusional” regions becomes softer than that of healthy tissue. Pioneering mechanotransduction studies have shown that soft substrates upregulate intermediate filament proteins in reactive astrocytes; however, many other aspects of astrocyte biology remain unclear. Here, we developed a platform for the culture of cortical astrocytes using polyacrylamide (PA) gels of varying stiffness (measured in Pascal; Pa) to mimic injury-related regions in order to investigate the effects of tissue stiffness on astrocyte reactivity and morphology. Our results show that substrate stiffness influences astrocyte phenotype; soft 300 Pa substrates led to increased GFAP immunoreactivity, proliferation, and complexity of processes. Intermediate 800 Pa substrates increased Aggrecan^+^, Brevican^+^, and Neurocan^+^ astrocytes. The stiffest 1 kPa substrates led to astrocytes with basal morphologies, similar to a physiological state. These results advance our understanding of astrocyte mechanotransduction processes and provide evidence of how substrates with engineered stiffness can mimic the injury microenvironment.

## Introduction

Traumatic brain injury (TBI) is a significant health concern across all age groups, spanning from children to young adults and the elderly population, all of whom are susceptible to TBIs, mainly as a result of falls. It can lead to a range of temporary to permanent sequelae, including psychosocial dysfunction, motor and cognitive deficits, and neurological disorders.^1^ TBI triggers a coordinated cascade of biochemical and molecular events that drastically modify the environment of the affected tissue, resulting in a dynamic state that evolves differently from healthy tissue over time. The rupture of the meninges, blood-brain barrier, and perineural nets allows for the entry of nonresident cells for debris clearance and the incorporation of fibronectin, laminin, and tenascin into the parenchyma, inducing dramatic changes in the extracellular matrix (ECM) composition, such as vimentin, GFAP, collagen IV, and laminin.^2,3^ ECM metalloproteases are activated to degrade the damaged tissue and promote neural plasticity.^4,5^ Local reactive astrocytes upregulate chondroitin sulfate proteoglycans (CSPG) to produce the glial scar and seal the injury.^4,6^ Together, these modifications significantly change the stiffness of the injured region, creating a distinctive microenvironment for neural regeneration and repair.^5^ Unlike other types of scars, glial scars in the murine cortex and spinal cord exhibit a softer consistency compared to healthy CNS tissue.^7^

Under basal-physiological conditions, cortical tissue ranges from 100 Pa to 1 kPa. However, after an acute penetrating injury, there is a significant decrease in elastic modulus, reducing to 50 Pa in the acute phase.^6^ These changes in matrix stiffness within the injury region may lead to the activation of cellular processes in anchorage-dependent cells, such as proliferation, migration, spreading, morphology, and stem-cell fate determination.^8−12^ Additionally, it may create a microenvironment that can be divided into at least three compartments with different mechanical properties and cell populations: a central soft contusion “core” region infiltrated by fibroblasts, inflammatory cells, blood vessels, and a various ECM proteins,^6^ an intermediate, peripheral or “pericontusional” region of glial scarring populated with newly proliferated and mature reactive astrocytes that delineate the border of the “core”, and finally, a stiff, secondary “pericontusional” region containing reactive microglia and remanent proliferative reactive astrocytes, which gradually stiffens as it approaches the healthy neural tissue.^2,4,13^

*In vitro* models can offer essential platforms to study the mechanical effects of matrix stiffness on cell behavior and phenotype changes. Biopolymer-based models, such as collagen I, collagen IV, fibronectin, laminin, and Matrigel, have been widely used to culture neural cells due to their biocompatible features.^14−17^ Hu et al.^18^ developed an *in vitro* alginate-collagen model with tunable properties to investigate the effects of matrix stiffening and softening on astrocyte response. This study showed that astrocytes plated on a soft substrate (50 Pa) showed a reactive response with increased intermediate filament (glial fibrillary acidic protein, GFAP) and inflammatory response (interleukin 1β, IL-1β) proteins, but when the substrate was stiffened (990 Pa), the reactive phenotype of the astrocytes was reversed to physiological levels. These findings suggest the need to carefully engineer models with suitable environments for mimicking areas and stages of the glial scar.

The challenges of studying cell behavior using biological materials include from batch-to-batch variability to the presence of specific ligands in the biopolymer that could trigger additional cellular responses.^19^ Furthermore, adjusting the polymer concentration is imperative due to the low mechanical properties of these biological materials. However, this adjustment may subsequently modify the gel surface, potentially affecting cell mobility.^20^ In contrast, polyacrylamide (PA) gels of various stiffnesses (measured in Pascal, Pa) can be controlled through titration of monomer and cross-linker, independent from any biomolecule. PA hydrogels are easily adjustable by varying acrylamide and bis-acrylamide concentrations and can simulate bone stiffness or very soft brain tissue.^21^ Due to their homogeneous surface topography and constant elasticity in all directions, they are considered a well-controlled system for studying the ability of neurons and astrocytes to sense and interact with the physical environment by converting mechanical stimuli into biochemical signals.^22,23^ In this study, we sought to investigate the effects of tissue stiffness on the astrocyte response following a traumatic brain injury (TBI). To this end, we engineered *in vitro* PA-gel-based microenvironment platforms to mimic the different stiffness within the injury region.

## Methods

### Animals

All protocols were approved by the Committee on Ethics in the Use of Animals of the University of California San Diego’s Animal Welfare Assurance (D16–00020, No. S17165). Jackson Laboratories (JAX) animal facility supplied isogenic C57Bl/6J pregnant mice (E14). Animals were housed in standard cages, with controlled light-dark cycles (12 h–12 h) and food and water ad libitum. All efforts were made to minimize suffering and the number of animals used.

### Primary Astrocyte Culture

The primary culture of astrocyte was adapted from Yang’s protocol,^24^ previously established in our lab.^25,26^ Briefly, we euthanized neonatal (P1) C57BL/6j mice by decapitation. Brain cortices were placed in HBSS solution (ThermoFisher, Boston, MA, USA), and meninges were removed. Cells were dissociated and homogenized with 0.25% trypsin (Corning, New York, USA) and 0.1% EDTA in HBSS, which was blocked with fetal bovine serum (FBS, Gibco, Grand Island, USA), and resuspended in astrocyte medium DMEM/F12 (Gibco, Grand Island, USA) containing 10% FBS, 1% GlutaMAX Supplement (200 mM glutamine; Gibco, Grand Island, USA) and 1% penicillin/streptomycin (100 IU; ThermoFisher, Massachusetts, USA). Finally, cells were plated in T25 flasks (ThermoFisher, Massachusetts, USA) and incubated at 37 °C in a CO_2_ incubator, with half-medium changes every 2–3 days. After 3 days, the flask was gently shaken to avoid microglia contamination, and the medium was changed. Cells were used between the first and fourth passages.

### Fabrication of Polyacrylamide (PA) Gel-Based Microenvironment Platforms

Three prepolymer solutions of different concentrations of acrylamide and the cross-linker bis-acrylamide (Sigma-Aldrich, St. Louis, USA) were prepared following the protocol Tse and Engler (2010):^21^ (a) 3% (w/v) acrylamide and 0.06% (w/v) bis-acrylamide; (b) 2% (w/v) acrylamide and 0.06% (w/v) bis-acrylamide; and (c) 2% (w/v) acrylamide and 0.05% (w/v) bis-acrylamide. Ammonium persulfate (APS; ThermoFisher, Massachusetts, USA) and *N*,*N*,*N*′,*N*′-tetramethylethylenediamine (TEMED; ThermoFisher, Massachusetts, USA) were added to the prepolymer solutions, and gels were allowed to polymerize for 30 min between a glass slide and a coverslip chemically modified with dichlorodimethylsilane (DCDMS; Sigma-Aldrich, St. Louis, USA). Sulfosuccinimidyl 6-(4′-azido-2′-nitrophenylamino)hexanoate (Sulfo-SANPAH, 0.2 mg/mL; Sigma, St. Louis, USA) was used to covalently bind proteins to the gel and activated with UV light (350 nm wavelength). Laminin 25 μg/mL (Gibco, Grand Island, USA) and 25 μg/mL poly-d-lysine (PDL; Sigma-Aldrich, St. Louis, USA) were added to the Sulfo-SANPAH and incubated overnight at 37 °C before plating cells. A 2D-conventional model (12 mm PDL and laminin-coated cover glasses) was used as a reference (control group) to compare the results.

### Atomic Force Microscopy Probing and Analysis

Atomic force microscopy (AFM) has become popular for evaluating the mechanical properties of cells, their components, and biomaterials.^27,28^ The AFM technique for measuring cell mechanics involves creating nanometer-scale indentations on a cell or substrate surface using a flexible cantilever equipped with a tiny, flexible microprobe. As the probe applies force to the surface, the cantilever deforms proportionately to the substrate’s deformation. A laser focused on the cantilever’s back detects this deformation by measuring the reflected light with a photodiode detector. This deflection data, recorded against the distance from the surface, forms a force–indentation curve, adjusting for deflection against an infinitely rigid substrate like glass. These curves can be analyzed with various models to determine the Elastic/Young’s modulus.^27,28^

### Cell Seeding in Polyacrylamide (PA) Gel-Based Microenvironment Platforms

For cell viability and immunocytochemistry assays, cells were seeded at a density of ∼50,000 cells/cm^2^ for analysis 3-days postseeding (3-dps, *n* = 3), representing acute injuries, and ∼25,000 cells/cm^2^ on the PA-gels and control group for analysis 7-days postseeding (7-dps, *n* = 3) representing chronic injuries. For proliferation assay and morphological analysis, ∼20,000 and 10,000 cells/cm^2^ were plated on PA-gels and control group 3- and 7-dps (*n* = 3), respectively.

### Controlled Cortical Impact Injury

Female C57BL/6J mice (8–12 weeks old, Jackson Laboratories) were used for the controlled cortical impact (CCI) model. Mice were anesthetized with 2.5% isoflurane, and buprenorphine analgesia was administered. A 5 mm craniotomy was performed over the right hemisphere between the bregma and lambda. The exposed brain was impacted using the ImpactOne instrument (Leica Biosystems) with a 2 mm diameter probe at a velocity of 3 m/s and a depth of 2 mm.

### Cell Viability

Viability assay was performed using the Live and Dead kit (ThermoFisher, Massachusetts, USA), where living cells were stained with calcein-AM (green fluorescence, 488 nm) and dead cells with ethidium homodimer-1 (red fluorescence, 640 nm). The protocol was performed according to the manufacturer’s instructions. Cells were incubated with the kit reagents and washed with PBS. Images were captured under the fluorescence microscope from at least five images in 20× magnification using the Fiji ImageJ software (1.53q, http://imagej.nih.gov/ij, USA). According to the manufacturer’s recommendation, nuclei were intentionally not counterstained. Both dead and live cells were counted to evaluate the total number of cells. Subsequently, we calculated the ratio of live cells to the total cell count to assess the viability.

### Immunocytochemistry

Briefly, all samples were fixed with 4% paraformaldehyde, washed with PBS, permeabilized with 0.1% Triton X, and incubated in a blocking solution containing 5% donkey serum (5% DS) for 60 min at room temperature. In sequence, samples were incubated with selected primary antibodies diluted in blocking solution added with 2 μg/mL fragment donkey antimouse (FAB; Jackson) overnight, at 2–8 °C. Primary antibodies: rabbit antiaggrecan (13880–1-AP, 1:500, ThermoFisher, Massachusetts, USA); rabbit antibrevican (PA5-PA5–118986, 1:500, Invitrogen, Massachusetts, USA); chicken antiglial fibrillary acidic protein, GFAP (ab4674, 1:500, Abcam, Cambridge, USA); mouse antineurocan (MA1–5843, 1:500, Invitrogen); rabbit anti-S100β (PA5–87474, 1:500, Invitrogen). The next day, samples were rinsed with 1× PBS and incubated with the corresponding secondary antibody (Table 2) in Hoechst 33342 solution (1:1000; Invitrogen, USA) for 45–60 min at room temperature. Secondary antibodies: IgG donkey antimouse Alexa 594 (715–005–150, 1:500, ThermoFisher, Massachusetts, USA); IgG donkey antichicken Alexa 594 (703–005–155, 1:500, ThermoFisher); IgG donkey antirabbit Alexa 488 (711–545–152, 1:500, ThermoFisher, Massachusetts, USA). Finally, samples were washed with 1× PBS and mounted onto slides with an aqueous solution (Fluoromount-G; Southern Biotech, Alabama, USA), and images were captured under a fluorescence microscope.

### Protein–Protein Interactions

We used STRING (v. 12.0) as an online free database server to predict interacting protein partners; this tool uses a combination of prediction approaches and sources (neighborhood, transferred neighborhood, gene fusion, co-occurrence, coexpression, experiments, databases, and text mining) to construct protein network maps.^29^ For Brevican and Ncan proteins, networks were made at a medium confidence level (0.400), with all active prediction methods and no more than ″10″ interactors. In the network, each node represents a protein and each edge represents an interaction; clusters were created using K-means clustering default options. Edge thickness represents the confidence of the prediction: low 0.150, medium 0.4, high 0.7, and highest 0.9. Finally, we created gene coexpression heat maps of a selected cluster to predict coexpressed gene partners. In the matrices, the color’s intensity indicates the level of confidence that two proteins are functionally associated (association score 0–1, low-high).

### Proliferation Assay

Cells were incubated with 20 μM EdU diluted in a prewarmed media (final concentration: 10 μM EdU) for at least 4h, as recommended by the manufacturer’s protocol (Click-iT EdU Cell Proliferation kit for Imaging, #C10337; ThermoFisher, Massachusetts, USA). Cells were fixed with 4% PFA for 15 min, washed with warm HBSS, and permeabilized with 0.5% Triton X and 3% bovine serum albumin (BSA; Sigma-Aldrich, St. Louis, USA). Cells were incubated for 30 min with the detection cocktail from the kit (1× Click-iT EdU reaction buffer, CuSO_4_, Alexa Fluor Azide, and 1× Click-iT EdU buffer additive) and protected from the light. Each well was washed with 1× PBS, and nuclei were counterstained with Hoechst 33342. Finally, the cover glasses were rinsed with 1× PBS and mounted onto slides with Fluoromount-G. Images were captured under the fluorescence microscope, and Hoechst (total cells) and EdU^+^ cells (newly proliferating cells) were counted from at least five images at 20× magnification using Fiji ImageJ software. The ratio EdU/Hoechst was calculated to assess the proliferation rate.

Three groups of solutions (*n* = 3 each) using different concentrations of bis- and acrylamide polymers were engineered to produce a hydrogel: (a) 2% (w/v) of acrylamide with 0.05% (w/v) of bis-acrylamide; 2% (w/v) of acrylamide combined with 0.06% (w/v) of bis-acrylamide; and last, 3% (w/v) of acrylamide with 0.06% (w/v) of bis-acrylamide. Hydrogels in a 6-well plate and covered in PBS were submitted to AFM (Asylum MFP-3D Atomic Force Microscope, Sanford Consortium for Regenerative Medicine). Elastic modulus values obtained with Asylum Research 13, Igor Pro 6.34 software from each hydrogel group were plotted on the graph (±SD error bars, *n* = 3).

### Image Acquisition

Images were captured with a Nikon Eclipse Ti2 microscope with a 10, 20, and 40× oil-immersion objective, fitted with a Hamamatsu Orca-Flash 4.0 digital camera, and processed using ImageJ software (1.53q, http://imagej.nih.gov/ij, USA).

### Image Analysis

#### Relative Fluorescence Intensity

Images of cells on PA-gel-based microenvironment platforms and control groups at 20× magnification were analyzed at selected time points (3- and 7-dps) (5–8 fields/image; *n* = 3). Channel images of the selected primary antibody (GFAP/S100β/ Acan/Bcan/Ncan) were transformed into 8 bits, and the corresponding mean gray values were measured. The relative fluorescence intensity analysis was used to quantify the cell protein content indirectly. Values of all groups were normalized by using the maximum-minimum method prior to statistical analysis.

#### Nearest Neighbor Distance (NND)

Images of cells on PA-gel-based microenvironment platforms and control groups at 20× magnification were analyzed at selected time points (3- and 7-dps; *n* = 3). NND analysis was used to measure and compare a population’s distribution pattern. The NND ratio was adapted from Clark and Evans methodology,^30^ which considers the relationship between the mean distance between the cells in a particular area and their relative density. Hoechst channel images were transformed into 8 bits; the threshold was used to remove outliers, and the “dilate” tool was used to create a new mask. Next, “centroid” and “area” were selected in the set measurements option, and the “analyze particles” tool (mask outlines) was used to display the *X* and *Y* coordinates (centroid) of the particle. Finally, we used the NND plugin from Fiji ImageJ software to get the nearest neighbor distances (https://icme.hpc.msstate.edu/mediawiki/index.phpNearest_Neighbor_Distances_Calculation_with_ImageJ.html). Values were used to calculate the number of cells analyzed, the cell density (p, particles/total area), the mean area of the particles, the mean distance (RA), and the relative density (, RE). The NND ratio corresponded to that of RA/RE. Ratios near zero describe an aggregated, clumped, and nonpatterned distribution, while ratios close to 2.15 describe a perfectly uniform and patterned distribution.

### Cell Counting

The “count cells” tool from Fiji ImageJ software was used to quantify: (a) the number of live and dead cells, (b) the number of GFAP^+^ for reactive astrocytes, (c) the number of cells (Hoechst or EdU for newly proliferating cells), (d) the number of Acan, Bcan and Ncan^+^ cells, and (e) the different morphologies in each image. The results of the ratios were presented as percentage values.

### Morphometric Analysis

Cells were seeded at a lower density to facilitate the visualization of their morphological features, with ∼20,000 and 10,000 cells/cm^2^ plated on PA-gels and control groups at 3- and 7-days postseeding (*n* = 3), respectively. Images in 20× and 40× of PA-gel-based microenvironment platforms and control group in the selected time windows (3- and 7-dps) were analyzed (30 images/*n*, *n* = 3). The plug-in simple neurite tracer (SNT) tool from Fiji ImageJ software was used to reconstruct and create a mask for the cell. Measurement options were set to quantify the following parameters: “area” (μm^2^), “convex hull” (μm^2^), “perimeter” (μm), “circularity” (area/perimeter ratio), “solidity” (area/convex hull area ratio). The same plug-in was also used to skeletonize the cells (10 images/*n*; *n* = 3) and calculate “number of branches” (un), “length” (μm), “intersections” (un), and “radius or distance from the cell body” (un) of the Sholl analysis. Sholl analysis is a methodological approach adapted from the work of Sholl and Uttley^31^ to characterize cell morphological features.

### Statistical Analysis

All experiments used three biological replicates (sample size, *n* = 3). Absolute (mean gray value, NND ratio, density, and mean distance) and relative values (proliferative response, proportion of S100β^+^, GFAP^+^, Acan^+^, Bcan^+^, and Ncan^+^ cells) were considered. For morphological analysis, absolute values were considered. Data was analyzed using one-way ANOVA followed by Tukey’s multiple comparison tests or two-way ANOVA for group analysis followed by Tukey’s multiple comparison tests. For all analyses, *p < 0.05, **p < 0.01, ***p < 0.001, and ****p < 0.0001 were considered statistically significant. The graphs show mean, standard deviation (SD), and standard error (SEM) depending on the case. Statistical analyses were performed using GraphPad Prism 9.0 software (San Diego, California, USA).

## Results

### Validation of PA-Gel-Based Microenvironment Platforms and Astrocyte Viability

Hydrogels using different bis- and acrylamide concentrations were engineered with different elasticity profiles matching Young’s ECM-injury region moduli.^6^ The profiles and regions considered were (a) ∼50 to 300 Pa at the lesion “core” and up to 150 μm from the injury *in vivo*, (b) ∼300 to 800 Pa at the ipsilateral “pericontusional” region and 150 to 400 μm from the injury *in vivo*,^6,18^ and (c) ∼1 kPa the basal-physiological environment.^6^

**Figure 1:**
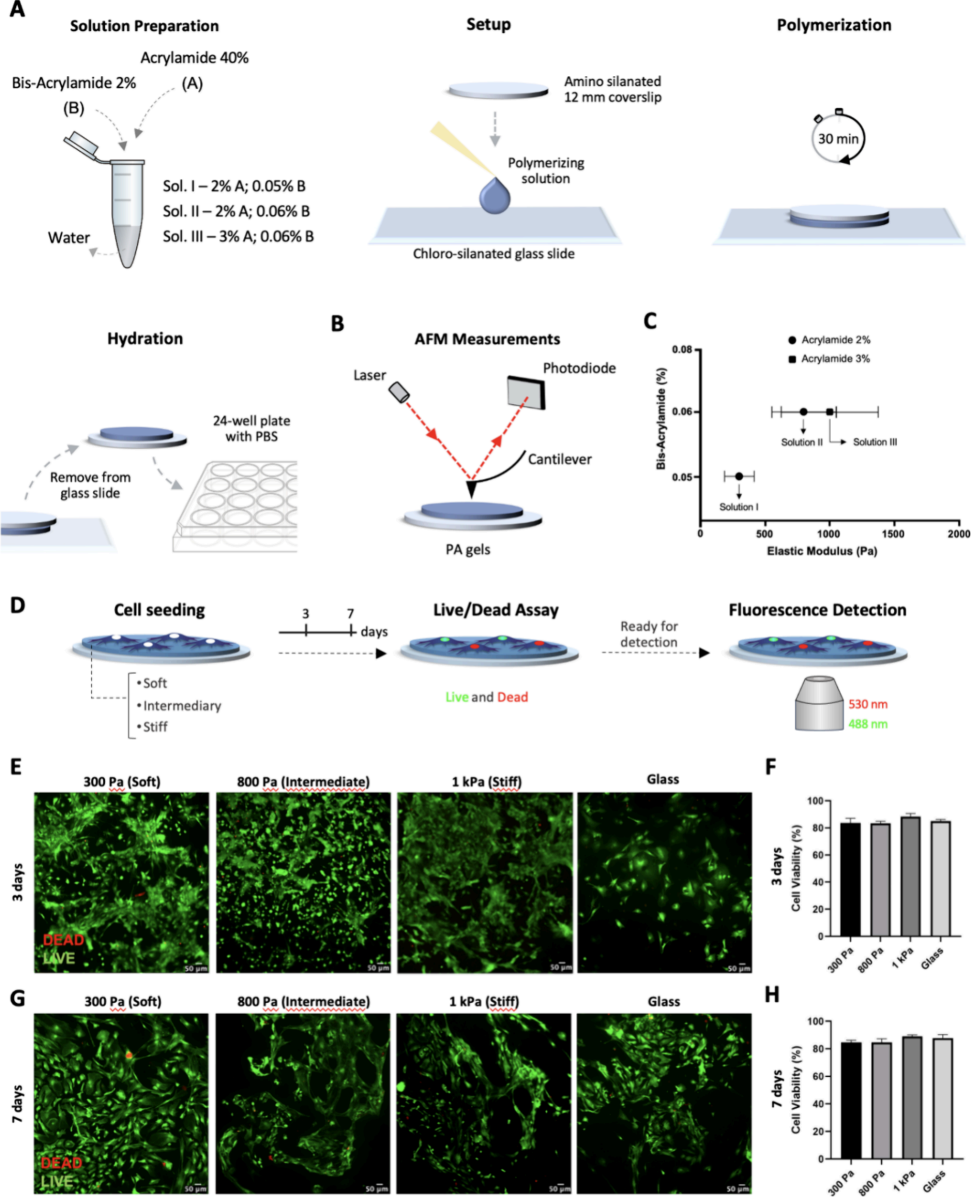
Scheme of Elastic Modulus evaluation of PA-gels and cell viability. (A) Scheme of acrylamide and bis-acrylamide combination to produce three solutions. The setup includes a presilanated coverslip, polymerizing solution, and chloro-silanated glass slide. Gel is allowed to polymerize between the coverslip and glass slide, and PBS is added for hydration. (B) Gel measurement using atomic force microscopy involves a sharp probe touching the gel surface, causing the cantilever to bend, which is then detected by changes in the laser light path read by a photodiode. (C) Graph of elastic modulus of the three hydrogels (±SD error bars, *n* = 3). The mechanical properties of the hydrogels were 300 Pa (soft), 800 (intermediate), and 1k Pa (stiff). (D) Scheme of primary astrocytes cultured on PA-gels and control group submitted to cell viability assay and analyzed 3- and 7-dps. (E) Representative images of live (green) and dead (red) cells on each group, 3-dps. (F) Graph bar shows quantitative analysis expressed in percentage of live cells, 3-dps (mean, ± SD error bars, *n* = 3). (G) Representative images of live (green) and dead (red) cells on each group, 7-dps. (H) Graph shows quantitative analysis expressed in percentage of live cells, 7-dps (mean, ± SD error bars, *n* = 3). Data was analyzed using one-way ANOVA followed by Tukey’s multiple comparison tests. Values with *p* < 0.05 were considered statistically significant. Scale bar 50 μm.

Acrylamide and bis-acrylamide were precisely combined to create three PA gel stiffnesses: soft, intermediate, and stiff, following Tse and Engler’s protocol.^21^ These solutions were polymerized between pretreated coverslips and glass slides (Figure 1A) and then hydrated for measurement using atomic force microscopy (AFM). A sharp probe detects surface properties by inducing bending, translating into measurements via deviation in the laser light path detected by a photodiode (Figure 1B). AFM measurements revealed that the PA-gel-based microenvironment platforms successfully mimicked the desired injury regions. Combining 2% (w/v) of acrylamide with 0.05% (w/v) of bis-acrylamide produced a soft gel of 300 Pa ± 113.9, mimicking the lesion “core”; 2% (w/v) of acrylamide combined with 0.06% (w/v) of bis-acrylamide, produced an intermediate stiffness of 800 Pa ± 249.5, the “pericontusional” region; and 3% (w/v) of acrylamide with 0.06% (w/v) of bis-acrylamide, the stiffer gel of 1 kPa ± 375.1, mimicking the physiological environment (Figure 1C).

On rigid substrates, such as glass (8 GPa), cells typically attach and spread easily across the surface.^18^ However, adhesion can be weakened on soft matrices, potentially compromising viability and altering morphology.^9,32,33^ As our PA-gels were very soft compared to glass, we used a combination of poly-d-lysine (PDL) and laminin to improve cell adhesion. PDL, a synthetic cationic polymer, enhances the interaction and attachment of anionic sites on the cells.^34^ Laminin, an abundant glycoprotein in the brain’s ECM, plays a crucial role in interacting with cell integrins and aquaporins, contributing significantly to the formation and migration of astrocytic processes.^35^

The cell viability was assessed 3- (3-dps, *n* = 3) and 7- days postseeding (7-dps, *n* = 3) using live and dead assays on PA-gels and the control group (Figure 1D). The groups showed no significant differences for both days (3- and 7 dps, Figure 1E), but all groups had more than 80% of live cells observed after a week (7-dps, Figure 1F). The data thus demonstrate that PA-gels-based microenvironment platforms ranging from 300 Pa to 1 kPa can provide a satisfactory environment for the primary culture of astrocytes.

### Soft PA-Gel-Based Microenvironments Triggers GFAP Expression on Astrocytes

**Figure 2:**
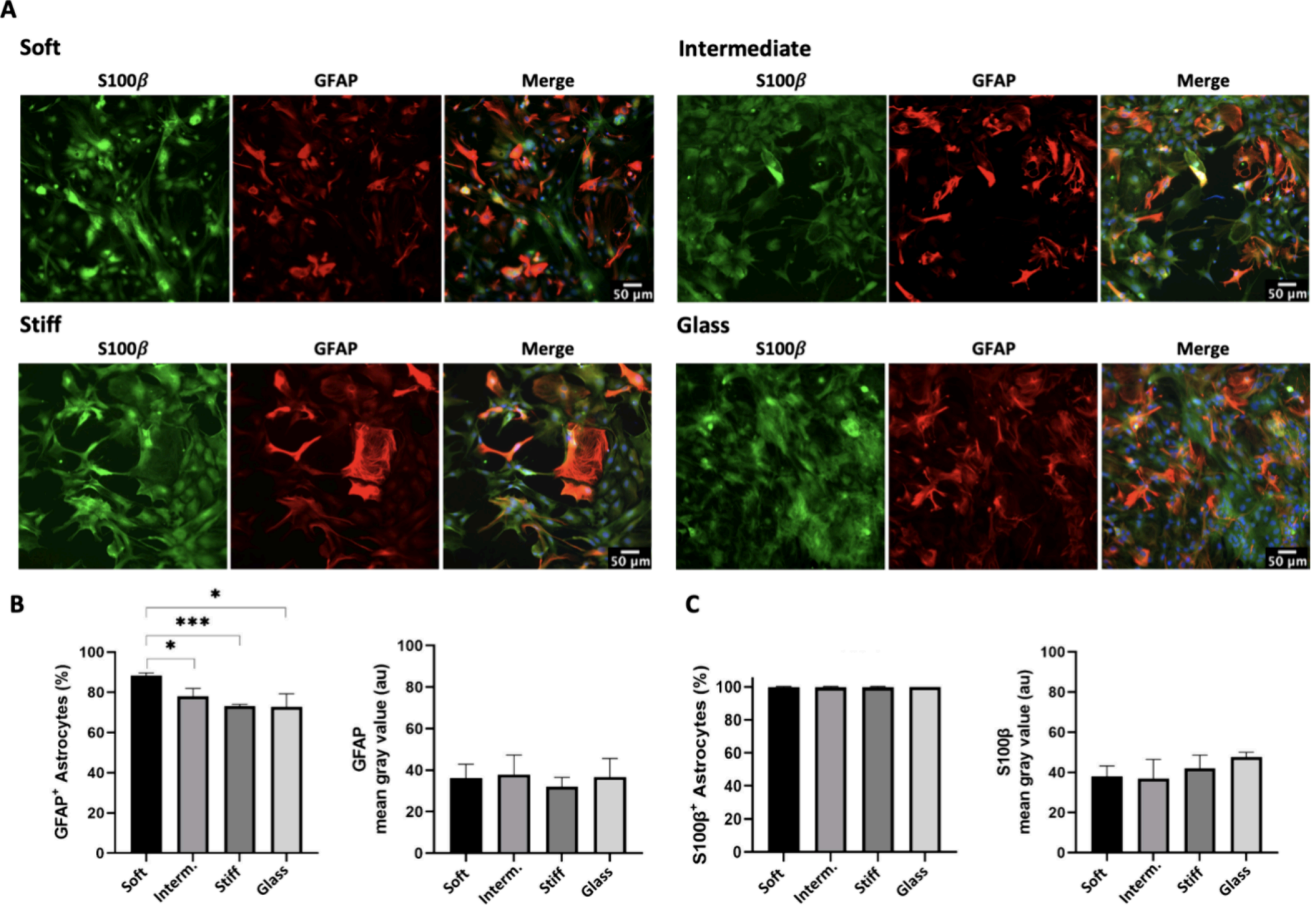
Astrocyte immunocharacterization and reactive response on PA-gel-based microenvironment platforms and glass (control group) 3-pds. (A) Representative images of GFAP and S100β immunolabeling. (B) Graph bar shows quantitative analysis of GFAP^+^ cells, expressed in percentage (mean, ± SD error bars, *n* = 3) and immunolabeling intensity (mean, ± SEM error bars, *n* = 3). (C) Quantitative analysis of S100β^+^ cells, expressed in percentage (mean, ± SD error bars, *n* = 3), and S100β immunolabeling intensity (mean, ± SEM error bars, *n* = 3). Data was analyzed using one-way ANOVA followed by Tukey’s multiple comparison tests. Values with *p* < 0.05 were considered statistically significant. Scale bar 50 μm.

On day three (3 dps), cortical astrocytes expressed GFAP and S100β in all PA-gels-based microenvironment platforms (Figure 2Aand Supplementary Figure 1). The analysis of GFAP immunolabeling revealed that an impressive 80% of the cells on the soft PA-gel were positive (GFAP^+^). This percentage was ∼1.2 times higher than the number of GFAP^+^ cells on the intermediate PA-gel (*p* < 0.014), stiff PA-gel (*p* < 0.0001), and the control group (glass, *p* < 0.016) (Figure 2B). Furthermore, the quantitative analysis of GFAP and S100β immunolabeling intensity (mean gray value) on astrocyte populations showed no statistical differences between the groups at 3 days postinjury (Figure 2B,C).

**Figure 3:**
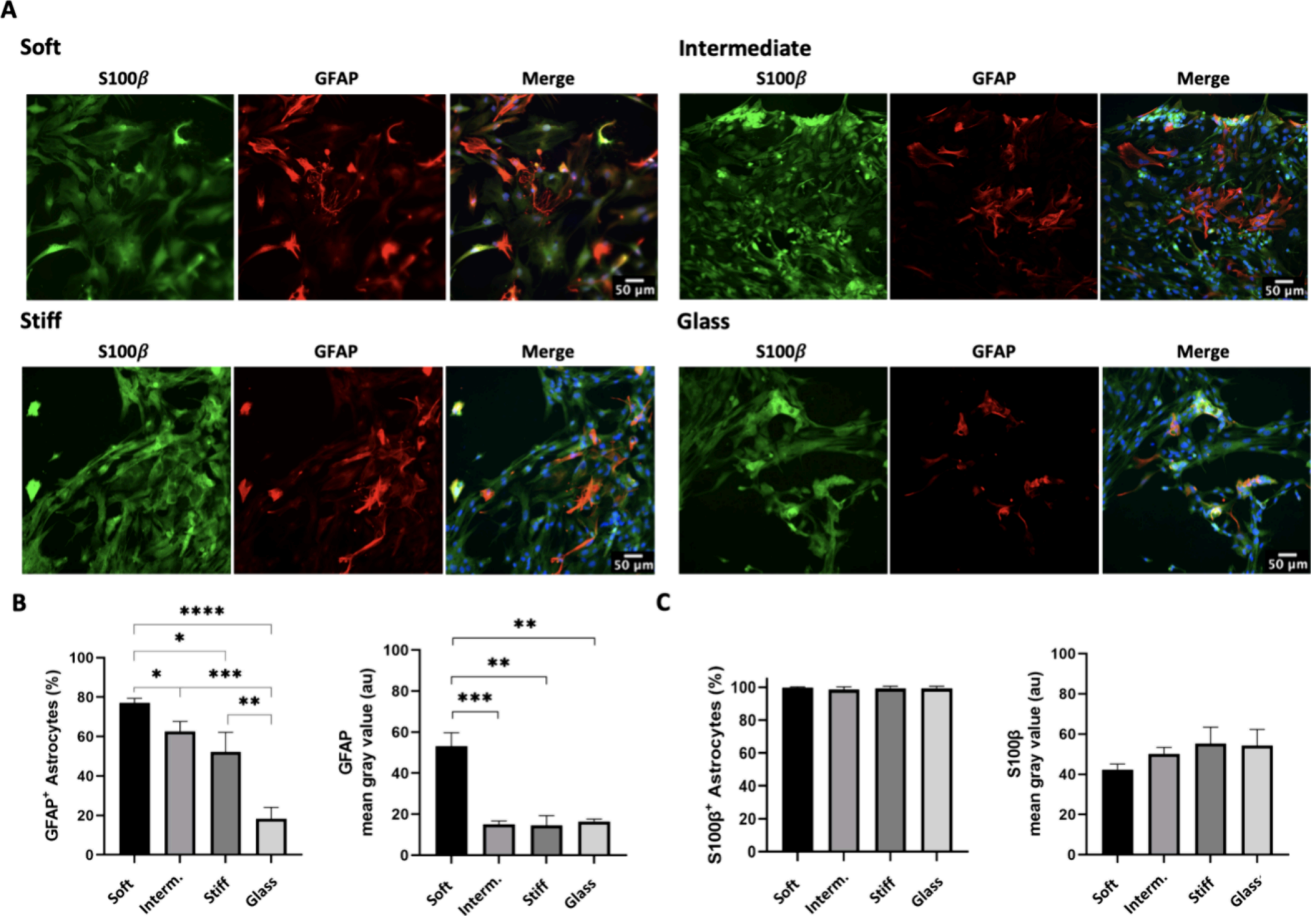
Astrocyte immunocharacterization and reactive response on PA-gel-based microenvironment platforms and glass (control group) 7-dps. (A) Representative images of GFAP and S100β immunolabeling. (B) Graph bar shows quantitative analysis of GFAP^+^ cells, expressed in percentage (mean, ± SD error bars, *n* = 3) and immunolabeling intensity (mean, ± SEM error bars, *n* = 3). (C) Quantitative analysis of S100β^+^ cells, expressed in percentage (mean, ± SD error bars, *n* = 3), and S100β immunolabeling intensity (mean, ± SEM error bars, *n* = 3). Data was analyzed using one-way ANOVA followed by Tukey’s multiple comparison tests. Values with *p* < 0.05 were considered statistically significant. Scale bar 50 μm.

On day seven (7-dps), cortical astrocytes expressed S100β in all PA-gel-based microenvironment platforms (Figure 3A and Supplementary Figure 1). However, for GFAP, there was a decline in the number of cells expressing this protein in the control group (glass), with ∼18% of GFAP^+^ cells observed (Figure 3B). This outcome aligns with the results of Xu,^36^ who reported 20% of GFAP+ cells after 1 week on glass. At 7-dps, the soft PA-gel exhibited a higher percentage of GFAP^+^ cells (∼78%). Furthermore, the fluorescence intensity of GFAP expression in the cells was also higher by ∼3.5 times than in other hydrogel groups (*p* < 0.0005 and *p* < 0.0015 comparatively to intermediate and stiff gel groups, respectively) and glass (*p* < 0.0006, Figure 3B). These findings prove that soft matrix strongly influences GFAP expression and astrocyte reactivation, even after 7-dps. The quantitative analysis of S100β^+^ cells and immunolabeling intensity (mean gray value) on astrocyte populations revealed no significant differences between groups 7-dps (Figure 3C).

### Proliferation Rate and Astrocyte Scar Border Behavior

The literature indicates that cell proliferation following brain damage occurs within 2 to 7 days in mice.^37,38^ Around the first 500 μm in an SCI model, more than 60% of the reactive astrocytes are proliferative, while less than 20% exhibit this phenotype up to 1200 μm from the lesion core.^4^ We questioned whether a mechanical stimulus could affect astrocyte proliferation as part of the astrogliosis response. We hypothesized that astrocytes plated on a gel mimicking the “core” region (soft PA-gel) would acquire a more proliferative phenotype compared to astrocytes on basal conditions (stiff PA-gel).

**Figure 4:**
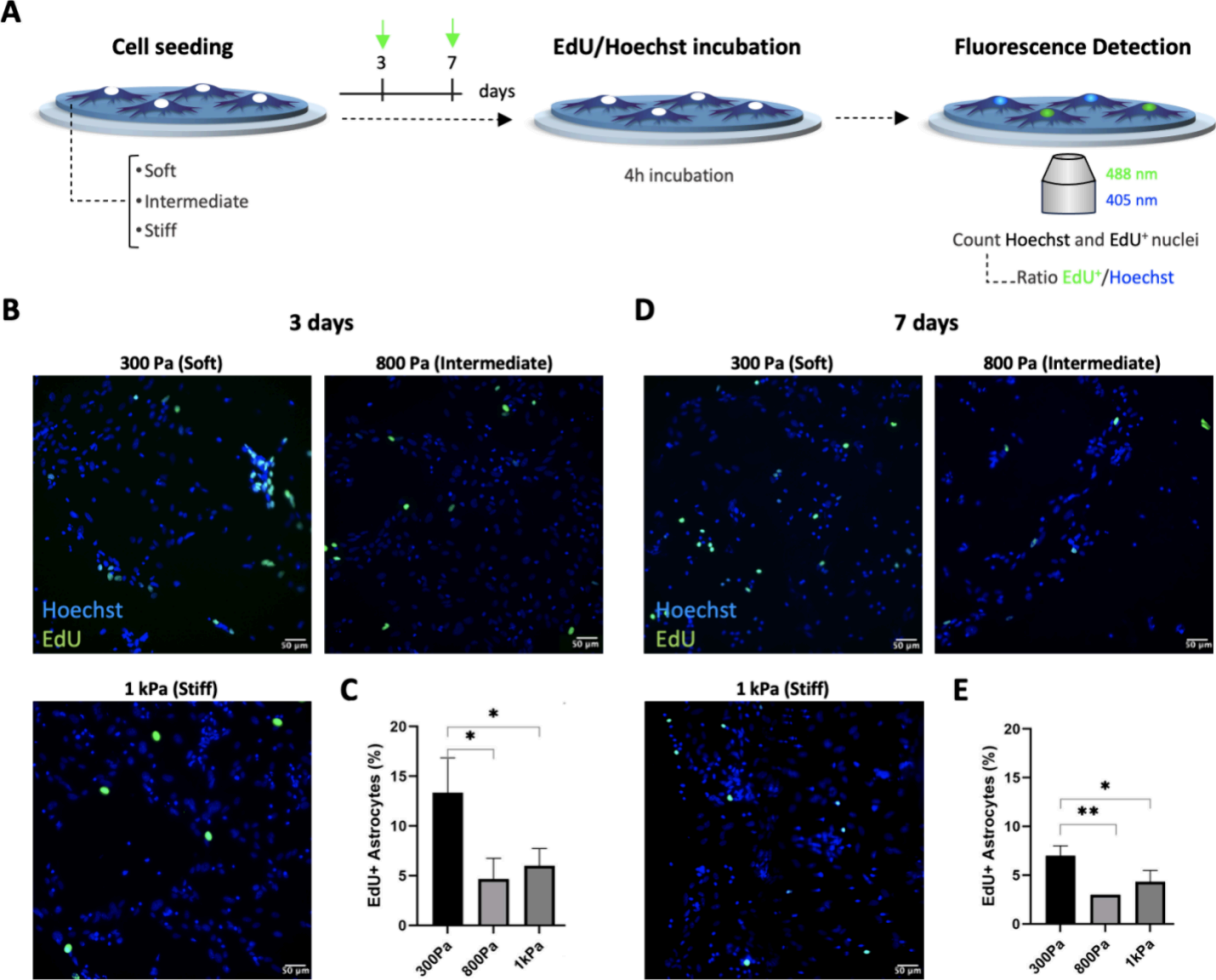
Proliferative response of astrocytes on PA-gels-based microenvironment platforms. (A) Scheme of primary cultures of astrocytes on PA-gels and analysis (3- and 7-dps). Astrocytes seeded on soft-, intermediate-, and stiff- PA-gels 3- and 7-dps. After incubation with EdU, cells were immunolabeled and counterstained with Hoechst. The ratio EdU^+^ (representing the newly proliferative cells)/Hoechst (representing the totality of cells) was measured to obtain the percentage of proliferative cells in groups. (B) Representative images of proliferation response after 3-dps. (C) Graph shows quantitative analysis of EdU^+^, 3-dps expressed in percentage (mean, ± SD error bars, *n* = 3). (D) Representative images of proliferation response, 7-dps. (E) Graph shows quantitative analysis of EdU^+^, 7-dps, expressed in percentage (mean, ± SD error bars, *n* = 3). Data was analyzed using one-way ANOVA followed by Tukey’s multiple comparison tests. Values with *p* < 0.05 were considered statistically significant. Scale bar 50 μm.

Astrocytes were incubated with EdU for 4 h and counterstained with Hoechst after being cultured 3- and 7-dps on PA-gels (Figure 4A). Counting nuclei stained with Hoechst fluorescence microscopy determined the total number of cells. The newly proliferating cells were identified by observing nuclei that were positive for EdU incorporation. Finally, we calculated the ratio of EdU-positive cells to Hoechst-stained nuclei to assess the proliferation rate. Our results indicated that for 3-dps (Figure 4B), 13% of the cells were proliferative on the soft PA-gel, significantly higher than the intermediate and stiff PA-gels (2.6 and 1.8 times higher, respectively, *p* < 0.021 and *p* < 0.031, Figure 4C). At 7-dps (Figure 4D), 7% of the cells were found to be proliferative on the soft PA-gel, significantly higher than the other hydrogels (2.3 times higher compared to intermediate PA-gel, *p* > 0.002, and 1.7 times higher compared to the stiff PA-gel, *p* > 0.03, Figure 4E).

**Figure 5:**
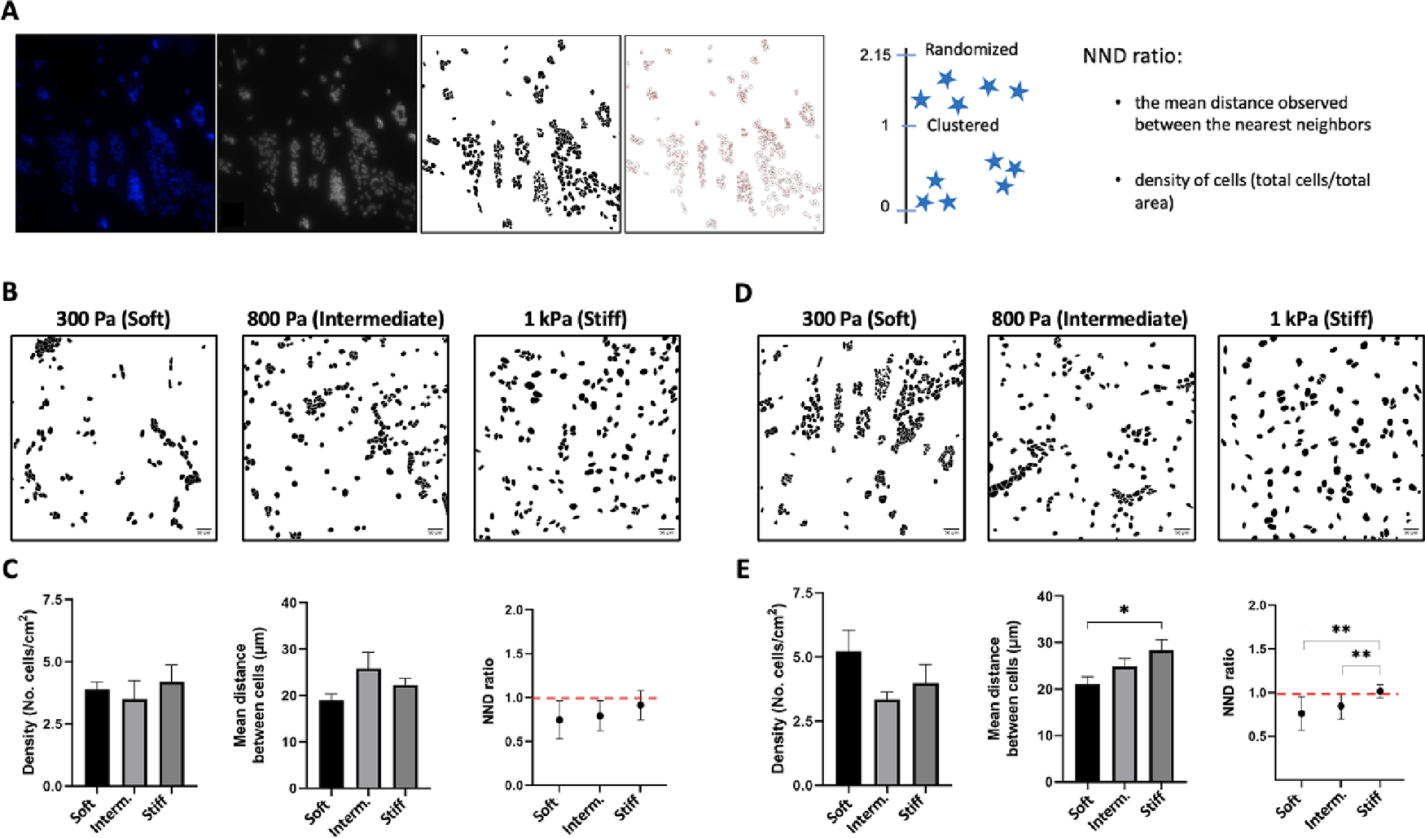
Analysis of cell population distribution considering density, mean neighbor distances, and distribution of astrocytes on PA-gel-based microenvironment platforms. (A) Representative images were transformed into masks and used for the near neighbor distance ratio (NND ratio) plug-in to measure the mean distance between cells and their density for response distribution. Results were categorized as aggregate, clumped, or nonpatterned when NND ratio was <1, and as uniform or patterned when NND > 1. (B) Representative images of the “masks” for each PA-gel group, 3-dps. (C) Graph bar of the density (mean, ± SEM error bars, *n* = 3), the mean distance between the cells (mean, ± SEM error bars, *n* = 3), and the NND ratio (mean, ± SD error bars, *n* = 3), 3-dps. (D) Representative images of the “masks” for each PA-gel group, 7-dps. (E) Graph bar of the density (mean, ± SEM error bars, *n* = 3), the average distance between the cells (mean, ± SEM error bars, *n* = 3), and the NND ratio (mean, ± SD error bars, *n* = 3), 7-dps. Data was analyzed using one-way ANOVA followed by Tukey’s multiple comparison tests. Values with *p* < 0.05 were considered statistically significant.

*In vivo,* this augmented proliferation rate of cells around the margin of lost neural tissue results in the emergence of an astrocyte scar border population. This population has a lower proliferation rate and aggregates around the injury site with overlapping branches, creating a barrier separating the viable neural tissue from the scar tissue.^4,13,39^ To further investigate whether mechanical stimuli could affect astrocytes to display a “scar border” distribution, we performed a nearest neighbor distance ratio (NND ratio) analysis. The NND ratio is a metric used to depict the distribution pattern of a population, with a lower NND ratio indicating a clustered, aggregated, and nonuniform distribution (Figure 5A). Our results showed that at 3-dps, astrocytes on all PA-gel groups tended to aggregate (NND ratio <1; Figure 5B,C), while at 7-dps, only astrocytes on the stiff PA-gel exhibited a uniform, patterned distribution (Figure 5D,E). The cell density measurement remained consistent across the groups. However, the mean distance between cells 7-dps was significantly higher in the stiff PA-gel group compared to the intermediate PA-gel (*p* < 0.004) and soft PA-gel (*p* < 0.001, Figure 5E). This result indicates that the mechanical stimulus directly affected the astrocytes’ spatial organization, explaining the observed distribution.

### Spatiotemporal Regulation of CSPG in Astrocytes Cultured on PA-Gel-Based Microenvironment Platforms

Following TBI, the expression of CSPG is altered and regulated to form the glial scar.^40,41^ This expression is modulated by inflammatory cytokines such as TNF-α, IFN-γ, IL-6, and IL-1β released at the injury site.^42^ However, the impact of mechanics on the expression of CSPG in the glial scar remains unexplored. A better understanding of the mechanisms underlying the formation of the glial scar could potentially lead to the discovery of new drug targets and the development of innovative regenerative strategies. Our inquiry focused on determining whether the stiffness of the PA-gels could influence astrocytes to produce CSPG, specifically, Acan, Ncan, and Bcan molecules. We assessed the astrocyte production of CSPG through mean gray value quantification.

**Figure 6:**
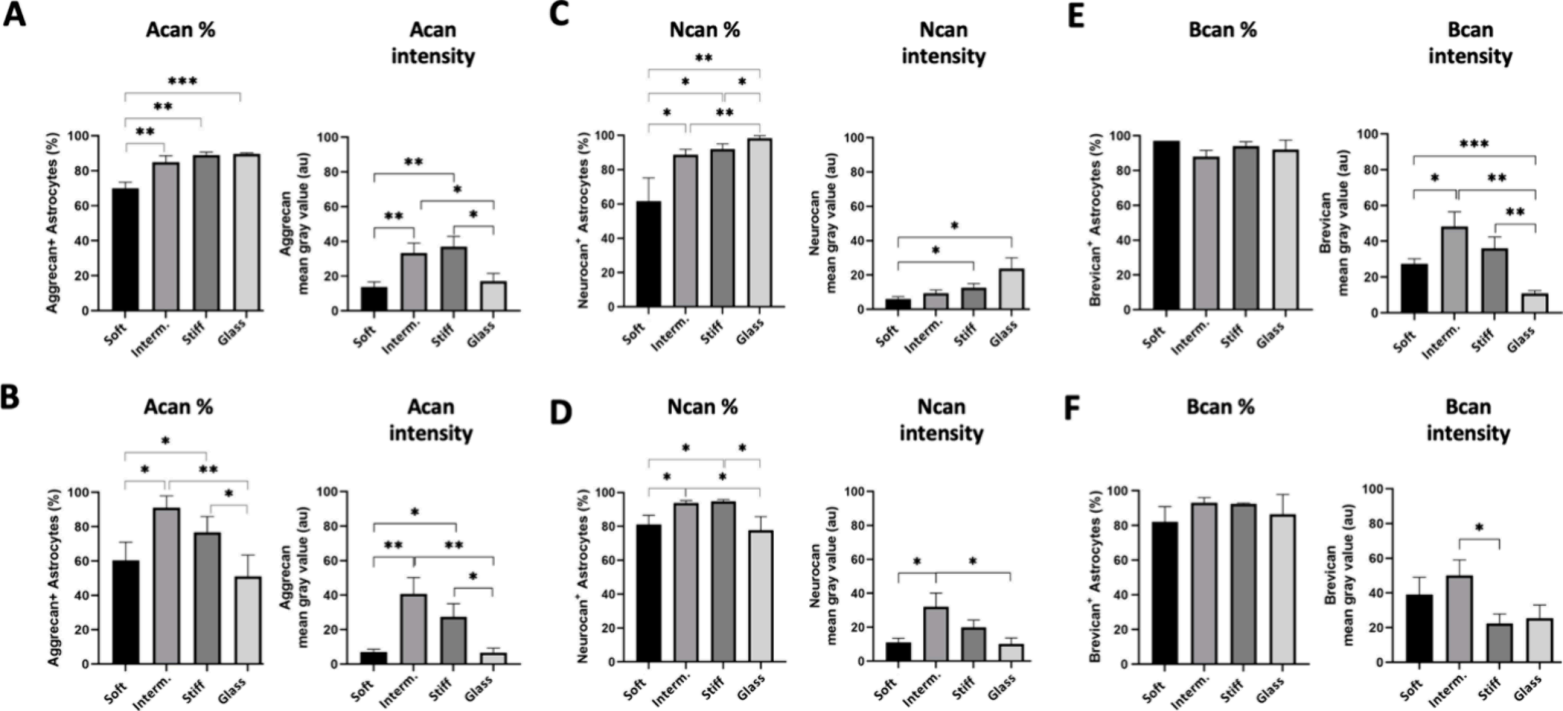
Evaluation of the response of astrocytes to express CSPG on PA-gel-based microenvironment platforms. (A) Graph shows a quantitative analysis of Acan^+^ (right), expressed in percentage (mean, ± SD error bars, *n* = 3), and Acan intensity (left) 3-dps (mean, ± SD error bars, *n* = 3). (B) Graph shows a quantitative analysis of Acan^+^ (right) expressed in percentage (mean, ± SD error bars, *n* = 3), and Acan intensity (left) 7-dps (mean, ± SD error bars, *n* = 3). (C) Graph shows a quantitative analysis of Ncan^+^ (right), expressed in percentage (mean, ± SD error bars, *n* = 3), and Ncan intensity (left) 3-dps (mean, ± SD error bars, *n* = 3). (D) Graph shows a quantitative analysis of Ncan^+^ (right), expressed in percentage (mean, ± SD error bars, *n* = 3), and Ncan intensity (left) 7-dps (mean, ± SD error bars, *n* = 3). (E) Graph shows a quantitative analysis of Bcan^+^ (right), expressed in percentage (mean, ± SD error bars, *n* = 3), and Acan intensity (left) 3-dps (mean, ± SD error bars, *n* = 3). (F) Graph shows a quantitative analysis of Acan^+^ (right), expressed in percentage (mean, ± SD error bars, *n* = 3), and Acan intensity (left) 7-dps (mean, ± SD error bars, *n* = 3). Statistical analysis was by one-way ANOVA with multiple comparisons of Tukey’s *posthoc*, and values with *p* < 0.05 were considered statistically significant.

Our findings indicate that over 85% of the cells in the intermediate and stiff PA-gel groups at 3-dps exhibited Acan expression, while the soft PA-gel group showed a significantly lower value (Figure 6A and shown in Supplementary Figure 3). The fluorescence intensity analysis further confirmed these results, showing that both the intermediate and stiff PA-gel groups had similar Acan values, ∼ 2.6 times higher than those observed in the soft PA-gel group (*p* < 0.007 and *p* < 0.002, respectively; Figure 6A and Supplementary Figure 3). Around 90% of the cells in the control group expressed Acan, comparable to those of the intermediate and stiff PA-gel groups. However, the mean gray value indicated a lower intensity than these groups, implying reduced Acan expression.

The percentage of Acan^+^ cells reached 91%, which was significantly higher than the soft PA-gel (*p* > 0.01) and the control groups (*p* < 0.08) by ∼1.5 and 1.8 times, respectively (Figure 6B). The stiff PA-gel also contributed to Acan expression, although to a lesser extent at 3-dps. The intensity quantification results were consistent with those of the Acan^+^ cells, showing the intermediate PA-gel ∼5.7 times more intense than the soft PA-gel (*p* > 0.007) and control group (glass, *p* > 0.008) and the stiff PA-gel ∼3.8 times more intense than the soft PA-gel and control group (glass, *p* > 0.03 for both; Figure 6B and Supplementary Figure 3). These findings strongly indicate that the intermediate PA-gel may be crucial in modulating astrocyte behavior and promoting ECM protein expression.

The most potent inhibitor of axon regrowth, Ncan, was found to be increased in pericontusional regions, ranging from 100 to 500 μm during the acute phase in a SCI model^43^ and in later stages in a TBI model.^41,44^ Thus, we hypothesized that Ncan would be upregulated in the intermediate PA-gels. Our findings showed that, following 3-dps, only 60% of astrocytes exhibited positivity for this proteoglycan when cultured on the soft PA-gel. This percentage was significantly lower than that observed for intermediate (*p* < 0.02) and stiff PA-gels (*p* < 0.01) and ∼1.6 times lower than the control group (glass, *p* < 0.009, Figure 6C and shown in Supplementary Figure 3). Interestingly, quantitative analysis of Ncan revealed a higher intensity in the stiff PA-gel group and glass, indicating that rigid substrates trigger the expression of this proteoglycan by astrocytes. The intermediate and stiff PA-gel groups produced 1.1 times more Ncan^+^ cells than the soft PA-gel (*p* < 0.02) and control groups (*p* < 0.01) after 7 days (Figure 6D and Supplementary Figure 3). As expected, the intensity analysis revealed that the intermediate PA-gel had the most expressive influence after 7-dps, increasing to 3.5 times Ncan immunolabeling in the astrocytes compared to 3-dps. The result represented an intensity 3 times higher than the soft PA-gel and control groups (*p* > 0.03, Figure 6D and Supplementary Figure 3).

Bcan is extensively expressed across various healthy regions of the brain.^45^ Approximately 90% of cells observed in PA-gel groups were Bcan^+^, 3- and 7-dps. The quantification of protein intensity on each PA-gel (mean gray value) varied, showing higher values in the intermediate and stiff PA-gel groups compared to the soft PA-gel (*p* < 0.04), and control group (glass, *p* < 0.002), 3-dps (Figure 6E and Supplementary Figure 3). Over time, the effects of the intermediate PA-gel on astrocytes were sustained and became significantly higher than those of the stiff PA-gel group, 7-dps (*p* < 0.02, Figure 6F and Supplementary Figure 3).

Bcan has two isoforms: it can be secreted into the extracellular space and play a role in cell signaling over longer distances, or it can be anchored to the cell surface, embedded in the membrane by a glycosylphosphatidylinositol produced through alternative splicing.^46,47^ According to Seidenbecher et al. (1995) and, more recently, Hußler et al. (2022), the upregulation of Bcan levels in the mechanically injured brain, epilepsy, and Small Vessel Diseases is not related to the glycosylfosfatidylinostiol isoform but soluble peptides instead.^46,48^ A closer analysis of Bcan immunolabeling showed the presence of tiny dots resembling vesicles (Supplementary Figure 4). The vesicles were initially observed on astrocytes in the intermediate PA-gel group 3 dps and became more prominent in both soft- and intermediate- PA-gel groups 7-dps. Although further analyses are required, soft- and intermediate-PA-gel groups may likely influence astrocyte production of Bcan variants, driving the cells to display these vesicle-like structures.

We then analyzed whether these vesicles could be visualized in *in vivo* tissue. Mouse brain tissues, obtained after 3- and 7- days postinjury, were processed and submitted for immunohistochemistry to analyze Bcan expression (Supplementary Figure 5). Bcan was detected in both the ipsilateral and contralateral regions. Interestingly, in contrast to our *in vitro* results, we observed the same pattern of structures exclusively in the ipsilateral region, proximal to the injury site.

### In Silico Bcan, Acan, and Ncan Protein Networks and Predictive Gene Coexpression Patterns

Next, in the context of our study, we explored *in silico* protein–protein networks and candidate gene coexpression partners of CSPG targets – Bcan, Acan, and Ncan – using STRING database, a bioinformatic tool that integrates text mining, wet-lab experiments, public databases, and genomic computational predictions to create dynamic protein–protein interaction graphs with edge confidence values.^29^ The analysis revealed associations between these CSPG and other proteins involved in astrocyte mechanotransduction processes.

**Figure 7:**
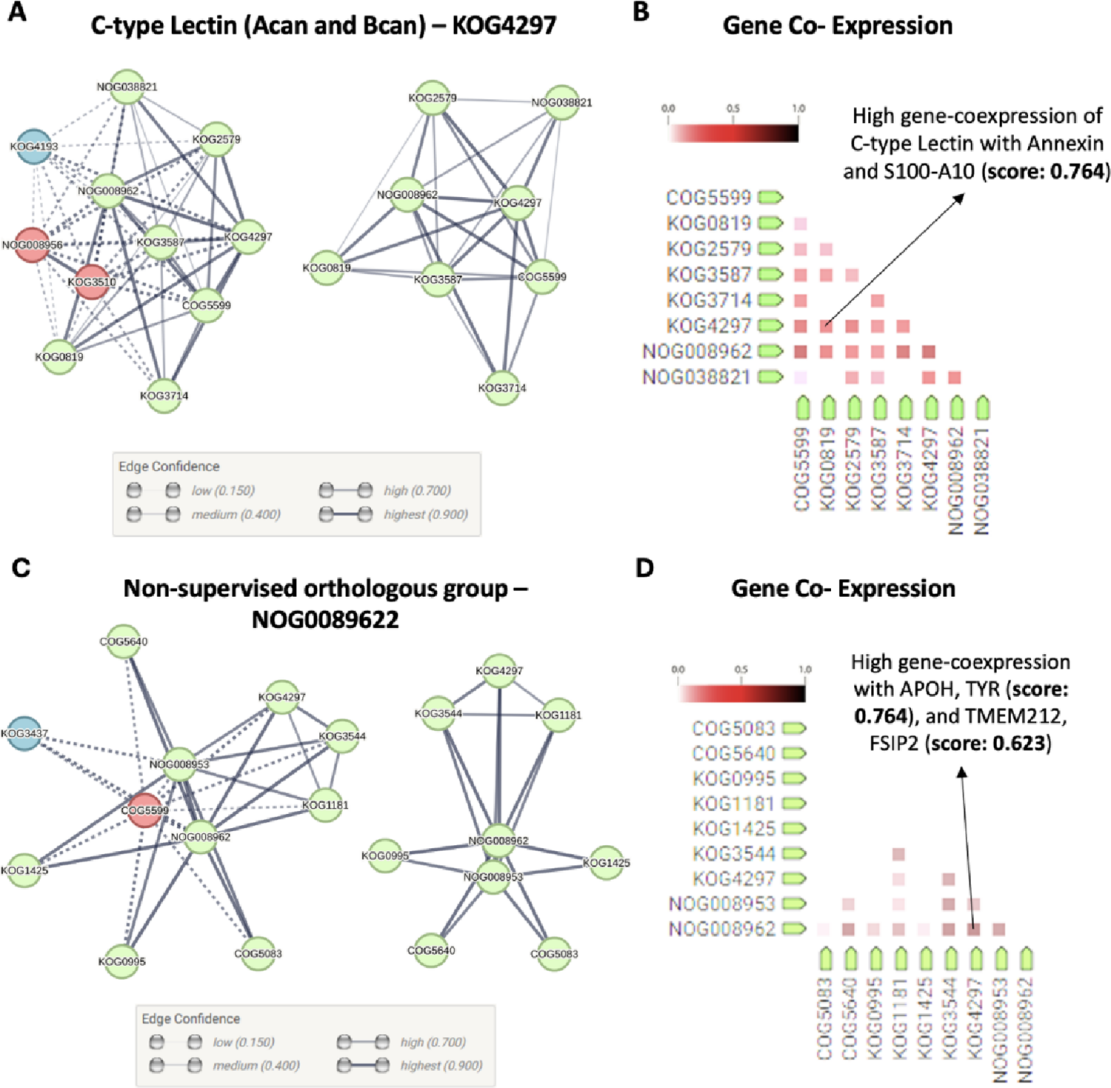
Bcan, Acan – C-type lectin core proteins, KOG4297 – and Ncan – nonsupervised orthologous group, NOG0089622 – interaction network and gene coexpression analyses. (A) Protein–protein network analysis displayed major clusters (green, red, and blue) for Bcan and Acan -C-type lectin family- proteins. Edges are represented as protein associations that are meant to be meaningful. Green cluster showed stronger and major protein–protein interactions. (B) Gene coexpression analysis displayed high association scores (AS) with Annexin A2 and S100-A10 (AS = 0.764). Abbreviations: COG5599: Protein Tyrosine Phosphatase; KOG0819: Annexin; KOG2579: Ficolin and related extracellular proteins; KOG3587: Galectin and Galactose-binding Lectin; KOG3714: Meprin A Metalloprotease; KOG4297: C-type lectin; NOG008962: nonsupervised orthologous group (comprehending the Ncan core protein); and NOG038821: nonsupervised orthologous group. (C) Protein–protein network analysis displayed major clusters (green, red, and blue). Edges represent protein–protein associations that are meant to be specific and meaningful, the green cluster showed stronger and major protein–protein interactions. (D) Gene coexpression analysis displayed high association scores (AS) with β2-glycoprotein 1 (APOH) and tyrosinase (TYR, AS = 0.764); and transmembrane protein 212 (TMEM 212) and fibrous sheath-interacting protein 2 (FSIP2, AS = 0.623). Abbreviations: COG5083: phosphatidate phosphatase PAH1; COG5640: secreted trypsin-like serine protease; KOG3544: collagens (type IV and type XIII); and KOG4297: C-type lectin (comprehending Bcan and Acan core proteins).

Human Bcan and Acan proteins, which belong to a broad conserved family of C-type -calcium binding- lectins (KOG4297), displayed substantial direct and indirect associations among these nodes. The most significant ones, each boasting an edge confidence level exceeding 0.700 (EC > 0.700), were with protein Tyrosine Phosphatase (COG5599, EC = 0.931), Annexin (KOG0819, EC = 0.902), Ficolin and related extracellular proteins (KOG2579, EC = 0.963), alectin and galactose-binding Lectin proteins (KOG3587, EC = 0.916), and Meprin A Metalloproteases (KOG3714, EC = 0.904, Figure 7A).

In the context of astrocytes, tyrosine phosphatases regulate focal adhesion and cytoskeletal proteins, impacting adhesion, migration, and stiffness sensing in astrocytes.^49,50^ Annexins influence membrane organization and signaling pathways, potentially participating in mechanotransduction.^51^ Metalloproteases, Meprin A,^52^ Galectins, and Lectins^53^ are involved in extracellular matrix remodeling and inflammation.

Gene coexpression analysis within this cluster showed a high significance association (score = 0.764) with C-type Lectin, Annexin A2, and S100A10 (KOG0819, Figure 7B). Human and mouse Ncan core proteins identified as the unsupervised orthologous group (NOG0089622) showed strong direct and indirect associations (EC > 0.700) with several proteins. These include Phosphatidate Phosphatase, PAH1 (COG5083, EC = 0.965), which, while not directly linked to mechanotransduction, may influence related pathways through its involvement in lipid signaling cascades that regulate cellular responses to mechanical stimuli.^54^ Additionally, the protein showed associations with secreted Trypsin-like Serine Protease (COG5640, EC = 0.970), which has not been directly associated with mechanotransduction but has been implicated in several cellular processes such as cell migration, proliferation, and astrocyte activation.^55^

Type IV and Type XIII Collagen (KOG3544, EC = 0.972) and C-type lectins, including Bcan and Acan core proteins (KOG4297, EC = 0.974, Figure 7C), may have implications in astrocyte mechanotransduction. Collagen receptors and their downstream signaling pathways in astrocytes could potentially sense and respond to mechanical stimuli from the surrounding extracellular matrix (ECM). Type IV and Type XIII collagen likely contribute to the mechanical properties of the ECM, influencing astrocyte behavior such as migration, proliferation, and differentiation in response to changes in tissue stiffness or mechanical stress.^56,57^

In the case of Ncan, mice gene coexpression analysis showed strong associations with 2-glycoprotein 1 (APOH) and Tyrosinase (TYR, AS= 0.764) as well as Transmembrane protein 212 (TMEM 212) and Fibrous sheath-interacting protein 2 (FSIP2, AS = 0.623, Figure 7D). While these associations did not directly involve proteins implicated in astrocyte mechanotransduction, it is noteworthy that Tyrosinase has been linked to changes in astrocyte morphology, transitioning from a polygonal shape to a stellate form.^58^

### PA-Gel-Based Microenvironment Platforms Enable Astrocytes with *In Vivo* Morphological Features

The existing literature indicates that 2D models often fail to accurately replicate the natural morphologies of astrocytes,^59,60^ thereby constraining our comprehension of their varied responses to physiological and pathological stimuli. The diverse shapes of cortical astrocytes in the brain, including stellate, perivascular, and bipolar forms, are inherently linked to their functional roles. Stellate has multiple branches emerging from the cell body; perivascular are analogous to the stellate shape but with endfeet in the extremities; and bipolar has two opposing processes, resembling the radial glia.^61^ In conventional culture models, astrocytes may exhibit atypical morphologies such as flattened shapes with extended processes or circular configurations lacking distinct branching patterns.^61^

Based on the literature description of these phenotypes, we further explored the influence of physical cues on astrocyte phenotype, 3- and 7-dps. As a control, we used a data set of astrocytes seeded on cover glasses, 3-dps.

**Figure 8:**
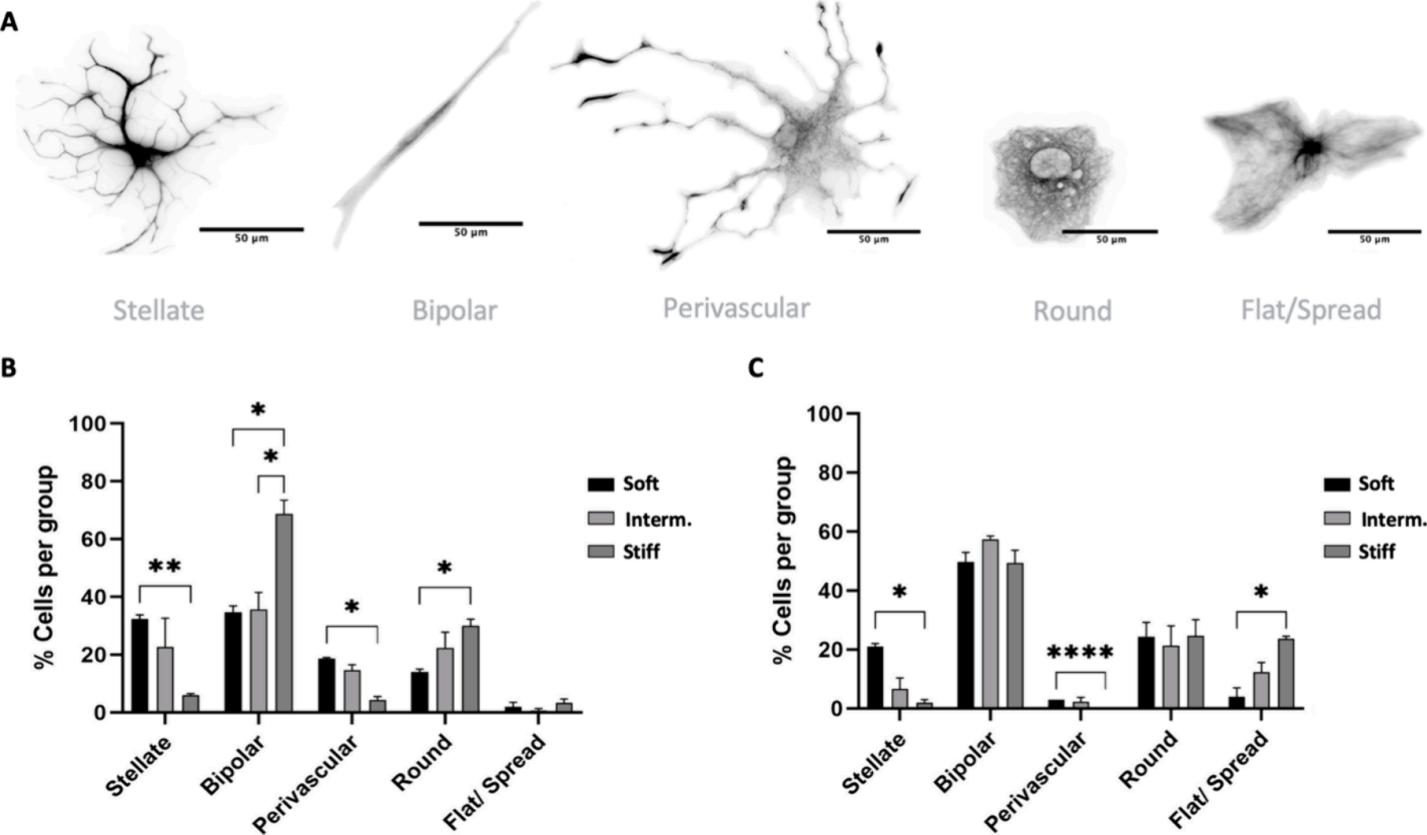
Distribution of astrocyte morphologies on PA-gel-based microenvironment platforms. (A) Representative images of the reconstructions of astrocytes in each PA-gel group. (B) Graph shows quantitative analysis of the morphologies in each group, 3-dps, expressed in percentage (mean, ± SEM error bars, *n* = 3). (C) Graph shows quantitative analysis of the morphologies in each group, 7-dps, expressed in percentage (mean, ± SEM error bars, *n* = 3). Statistical analysis was by two-way ANOVA (using “stiffness” and “morphologies” as factor 1 and 2, respectively) with multiple comparisons of Tukey’s *posthoc*, and values with *p* < 0.05 were considered statistically significant. Scale bar 50 μm.

Astrocyte morphologies were reconstructed using GFAP staining and the SNT software tool to help the characterization (Figure 8A and Supplementary Figure 6 for more details of stellate/perivascular and round/flat). Results revealed that bipolar cells, a natural morphology, constituted a significantly higher proportion of astrocytes on the stiff PA-gel (68%) compared to both soft (*p* < 0.04) and intermediate PA-gel groups (*p* < 0.03) (Figure 8B). In contrast, stellate cells, the morphology most commonly observed *in vivo*,^61^ comprised 32% of astrocytes cultured on the soft PA-gel group, demonstrating a marked increase of 5.4 times compared to the stiff PA-gel group (3-dps, *p* < 0.009). This percentage decreased to 21% after a week (7-dps); however, this group still had the highest number of stellate cells, with approximately 10 times more than the stiff PA-gel group (*p* < 0.01) and 3 times more than the intermediate PA-gel group, 7-dps (Figure 8C). In addition, in soft PA-gels, perivascular cells represented 18% of the culture, while in intermediate PA-gels, they comprised 15%, and in stiff PA-gels, they constituted 4% in stiff PA-gels at 3-dps (Figure 8B). Although these percentages decreased after 7 dps, they could still be observed in soft and intermediate PA-gel groups (Figure 8C).

The stiff PA-gel group predominantly showed round morphologies at 3-dps, with 30% of astrocytes displaying this morphology, 2-fold higher than the soft PA-gel group (*p* < 0.01, Figure 8B). At 7 dps, no significant difference was observed between these two groups (Figure 8C). Notably, at 3-dps, only 3% of the astrocytes displayed a flat spread morphology in the PA-gels (Figure 8B). At 7-dps, this morphology increased by 23% in the stiff PA-gel group, reaching approximately 6-fold more than the soft PA-gel group (*p* > 0.04, Figure 8C).

### Morphological Complexity of Astrocytes Plated on PA-Gel-Based Microenvironment Platforms

**Figure 9:**
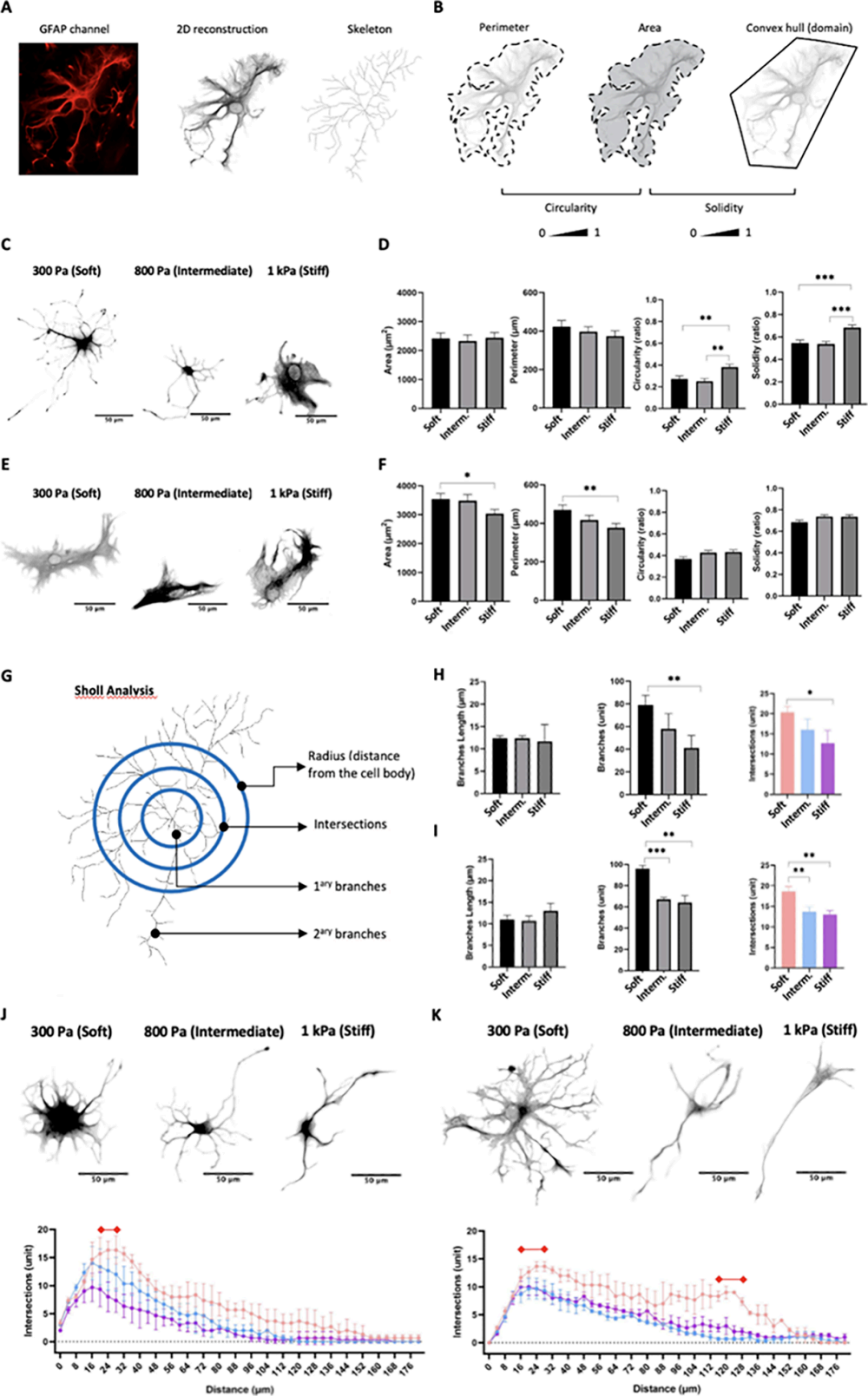
Morphometric analysis of astrocytes on PA-gel-based microenvironment platforms. (A) GFAP channels were converted into 8-bits images and, using SNT plug-in, 2D reconstructions and skeletons of the cells were generated. (B) Morphometric analyses using 2D parameters: perimeter, area, and convex hull area. These values estimate circularity, area, and solidity, respectively. (C) Representative images of 2D reconstructions of astrocytes highlight the morphological differences on each substrate 3-dps. (D) Graphs show quantitative analysis of 2D morphological parameters (area, perimeter, circularity, and solidity) to evaluate astrocyte response to each PA-gel 3-dps (mean, ± SEM error bars, *n* = 3). (E) Representative images of 2D reconstructions of astrocytes highlighting the morphological differences on each substrate 7-dps. (F) Graphs show quantitative analysis of 2D morphological parameters (area, perimeter, circularity, and solidity) to evaluate astrocyte response to each PA-gel 7-dps (mean, ± SEM error bars, *n* = 3). (G) Scheme of Sholl analysis employed to assess cell complexity by determining intersections, primary and secondary branches, and the distance (radius*) from the cell body. (H) Morphometric quantification of process length and the number of branch points, 3-dps (mean, ± SD error bars, *n* = 3). (I) Morphometric quantification of process length and the number of branch points, 7-dps (mean, ± SD error bars, *n* = 3). (J) Representative images of 2D reconstructions of astrocytes highlighting the amount of primary and secondary branches on each substrate 3-dps. Graphs of the number of intersections and intersections relative to the distance (graph of complexity) show the number of branches distributed along the astrocytes, 3-dps (mean, ± SEM error bars, *n* = 3). Red bar represents a peak of intersections at that distance from soma. (K) Representative images of 2D reconstructions of astrocytes highlighting the amount of primary and secondary branches on each substrate 7-dps. Graphs of the number of intersections and intersections relative to the distance (graph of complexity) show the number of branches distributed along the astrocytes, 7-dps (mean, ± SEM error bars, *n* = 3). Red bars represent peaks of intersections at that distance from soma. *Radius from soma represents 4 μm concentric grids centered on the cell soma. Statistical analysis was by one-way ANOVA with multiple comparisons of Tukey’s *posthoc* for parameters evaluation, and two-way ANOVA with multiple comparisons (using “intersections” as factor 1 and “distance” as factor 2) of Tukey’s *posthoc* for complexity graphs values with *p* < 0.05 were considered statistically significant. Scale bar 50 μm.

In order to gain a comprehensive understanding of astrocytes’ heterogeneity, it is essential to investigate how modifications in the injured microenvironment influence their morphological complexity. To this end, we aimed to quantify the previously observed morphologies and assess their association with astrogliosis. To achieve this, we employed the SNT plug-in to reconstruct (Figure 9A) and quantify 2D morphological parameters – perimeter, area of the cell body, and domain area (convex hull area) – for each cell (Figure 9B). Subsequently, we calculated the descriptors, circularity, and solidity to assess the cell’s shape by comparing the perimeter-to-area ratio (with values close to 1 indicating a perfect circle) and the area-to-domain ratio (with values close to 0 indicating a tendency to form intricate, mesh-like structures; Figure 9B).^62,63^

At 3-dps, no significant differences were observed between the PA-gel groups regarding area or perimeter. However, the results of circularity and solidity indicated that astrocytes on the stiff PA-gel were significantly more circular and less complex compared to those on the soft (*p* > 0.003 for circularity; *p* > 0.0002 for solidity) and intermediate PA-gel groups (*p* > 0.0004, for circularity; *p* > 0.0002, for solidity, Figure 9C,D). At 7-dps, the soft and intermediate PA-gel groups displayed similar results to those in the stiff PA-gel group regarding circularity and solidity. Additionally, astrocytes in the soft PA-gel demonstrated significantly larger area and perimeter than those in the stiff PA-gel group (*p* > 0.04 and *p* > 0.006, respectively; Figure 9E,F). These parameter alterations from day 3- to 7-dps (Figure 9D,F, respectively) may be attributed to the increased number of branches, leading to a larger area and rounded shape.

Subsequently, we conducted a Sholl analysis to investigate the correlation between morphological features and the astrocyte reactivation response. Arbor complexity was assessed by measuring the length, counting the number of the primary and secondary branches, and determining the number of intersections (Figure 9G). Astrocyte branches from 3- and 7-dps in all PA-gel groups exhibited a similar length. However, the number of branches was ∼1.5 times greater on the soft PA-gel 3-dps, and number of intersections were ∼1.7 times more than those from the stiff PA-gel group (*p* < 0.009 and *p* < 0.02, respectively), indicating that the softness of the PA-gel leads to the enhancement of complex structures (Figure 9H). At 7-dps, astrocytes displayed ∼1.5 times more branches and ∼1.4 times more intersections on the soft PA-gel compared to the intermediate (*p* > 0.0002 and *p* > 0.006, respectively) and stiff PA-gel groups (*p* > 0.001, and *p* > 0.003, respectively) at 7-dps (Figure 9I). These results validate the significant influence of PA-gel softness on the astrocyte morphological response.

The graph illustrating the intersection/distance correlation provides insights into the spatial distribution of the arbor complexity. It shows where branches are concentrated concerning the cell structure, whether near or far from the cell body. In all experimental groups, a spike of intersections (indicated by the red bar on the graph) was observed at a distance of 20–28 μm (mean of 24 μm, SD ± 4 μm) from the cell body (Figure 9J). Astrocytes from the soft and intermediate PA-gel groups exhibited a comparable response at this peak. However, astrocytes cultured on the soft PA-gel group displayed a significantly higher number of branches near the cell body compared to the stiff PA-gel group (*p* < 0.03 at 20 μm, and *p* < 0.02 at 24 and 28 μm). Moreover, the lines overlapping at 7-dps on the graph represent the intermediate (blue line) and stiff PA-gel groups, indicating a transition in astrocyte response: those cultured on the intermediate PA-gel shifted their behavior from resembling those on the soft PA-gel to resembling those on the stiff PA-gel group (Figure 9K).

After a week (7 days), we confirmed the presence of a peak of complexity (branches enrichment represented by the red bars in the graph) near the cell soma, around 16–32 μm (mean 24 μm, SD ± 8 μm) in all PA-gel groups (Figure 9K). Notably, the astrocytes on soft PA-gels exhibited a distinct second peak, occurring at approximately 116–128 μm (mean 122 μm, SD ± 6 μm). This second peak revealed astrocytes with more secondary branches further from the cell soma compared to the intermediate and stiff PA-gel groups (*p* < 0.03 and 0.02 at 116 μm; *p* < 0.02 and 0.003 at 120 μm; *p* < 0.004 and 0.04 at 124 μm; and *p* < 0.004 and 0.01 at 128 μm respectively; Figure 9K).

## Discussion

In this study, we explored how astrocytes respond to TBI, considering both the injury severity and proximity to the injury core. Our engineered *in vitro* PA-gel-based microenvironment platforms simulated varying stiffness within the injury region, aiming to understand the link between mechanical stimuli and astrogliosis.

PA gel models, known for accessibility and well-documented nature, enable precise control over mechanical properties, which is ideal for studying astrocyte mechanotransduction.^21^ Unlike biopolymers, they offer unique advantages due to their precise control over the mechanical properties. Their ability to adjust stiffness through simple titration of monomer and cross-linker concentrations facilitates replication and validation across laboratories.^64^ Additionally, the homogeneous surface and consistent elasticity of PA-gels create an ideal environment for studying astrocyte mechanotransduction.^64^ These platforms successfully matched Young’s moduli post-TBI, maintaining high cell viability over 7 days.

We employed common biomarkers, GFAP and S100β, to evaluate the reactive astrocyte responses. GFAP, a type-III intermediate filament, is typically upregulated and found in both the lesion core and peripheral area, serving as a marker for astrocyte reactivity.^2,65−68^ The accumulation of S100β occurs in astrocytes and has been detected in the blood of individuals with severe TBI.^69−71^ In our investigation into the connection between mechanical cues and the expression of GFAP and S100β, based on previous studies that utilized gelatin and alginate scaffolds,^18^ we observed that the soft PA-gel, designed to resemble the injury core, notably influenced GFAP expression. Specifically, it sustained a reactive state in most cells over a week.

In contrast, results from the astrocytes cultured on glass (control group) were consistent with Xu’s data,^36^ where fewer than 20% of GFAP^+^ cells were present in the culture after 1 week (7-dps). That led to our hypothesis that the traditional 2D model may not provide an ideal environment for studying the effects of astrogliosis. Notably, intermediate and stiff PA-gel groups resembled *in vivo* healthy GFAP^+^ astrocyte percentages described in the literature (∼60%),^72^ indicating suitability for mimicking healthy neural tissue.

Apart from the upregulation of GFAP, mechanoreceptors, such as the ion channel Piezo1 and connexin 43 (found in gap junctions and hemichannels), can initiate other astrogliosis responses, such as migration and proliferation.^73^ Typically, mature astrocytes do not exhibit proliferative properties.^74^ However, these channels open in response to injuries and mechanical stimulation, facilitating an excessive Ca^2+^ and ATP exchange to neighboring cells. This activation of astrogliosis influences migration and proliferation.^75−80^ Moreover, substrate stiffness can induce proliferation through activated integrins, initiating focal adhesion kinases and triggering pathways that regulate proliferation via ERK activation.^81,82^

Astrocyte proliferation in response to brain damage correlates with the severity and proximity to the “core″. At the peripheral “pericontusional” region of the lesion (between 150 and 400 μm) or in minor injuries, glial cells display a mild reactivity, with a limited number of GFAP^+^ cells reacquiring a proliferative phenotype.^2,26,68,83−85^ In contrast, at the “core” or in situations involving severe injuries such as traumatic wounds, hypoxia, inflammations, infectious diseases, and neurodegenerative conditions, both GFAP level proliferation rate increases.^65^*In vitro*, the baseline proliferation rate of astrocytes cultured on a traditional culture system (glass) is 5%.^74^ Our results showed that the soft PA-gel significantly influenced astrocyte proliferation.

Our NND ratio analysis revealed that astrocytes cultured on soft and intermediate PA-gel substrates tended to aggregate, as indicated by a clumped distribution. Our analysis centered on assessing the aggregation of cells within the context of astrogliosis, utilizing the nucleus mask and its coordinates for simplified visualization. While this approach provided valuable insights, alternative methods, such as staining the entire cell with cytoskeleton markers, such as phalloidin^86^ or vimentin,^87^ and determining centroid coordinates could offer complementary perspectives. These findings shed light on the mechanisms orchestrating astrocyte responses to mechanical cues and can potentially guide future investigations into the dynamic processes of glial scar formation and tissue repair.

Another aspect of astrogliosis is the regulation of CSPG expression, including Acan, Bcan, and Ncan. The spatiotemporal changes in CSPG expression following traumatic events are closely linked to the brain tissue mechanics in pathological conditions, and understanding these mechanisms can be crucial for developing new therapies and drugs. *In vivo,* events like CCI event results in a reduction of Acan expression at the lesion core due to the mechanical disruption of perineural nets and the activates metalloprotease, which soften the area during the onset of the inflammatory phase.^88−91^ Conversely, Acan level increases in the pericontusional region, where it is expressed by astrocytes and oligodendrocytes.^41^ Similarly, Ncan levels are upregulated in pericontusional areas during the chronic phase in both SCI and TBI models.^41,43,44^ TBI and SCI activate A Disintegrin and Metalloproteinase with Thrombospondin motif (ADAMT) enzymes, leading to increased Bcan proteolytic products at the lesion core and pericontusional region, peaking at 1 and 2 weeks postinjury, respectively.^42,48,92^ In summary, the degradation of CSPG in the affected tissues by metalloproteases, along with their upregulation in the surrounding areas, plays a critical role in regulating tissue mechanics following traumatic injuries.

Interestingly, the immunofluorescence assay showed small Bcan aggregations, suggesting the presence of Bcan vesicles – like in intermediate and soft PA-gels after 1 week. It is known that Bcan has two isoforms: one, with glycosylfosfatidylinostiol that binds to the hyaluronic acid and conducts the mechanical signaling,^93−95^ while the other, a soluble isoform, that is highly produced during neurodegenerative diseases and post-trauma inflammation, serving as a signal to cells away from the lesion core.^96,97^ The existence of these vesicle-like structures in gels ranging from 300 to 800 Pa (soft and intermediate PA-gels) suggests a potential role for Bcan in triggering post-traumatic signaling pathways, highlighting the need for additional research into mechanotransduction roles in neurological diseases.

This work also sheds light on potential CSPG partners and protein–protein networks relevant to traumatic injuries. Interactions within the brain core matrisome and extracellular matrix (ECM)-affiliated proteins have far-reaching effects on both biochemical and biomechanical aspects, influencing critical biological processes such as morphogenesis, maturation, homeostasis, and the progression of neurodegenerative and brain damage conditions.^98^ In our *in silico* analysis, the coexpression of Bcan and Acan with Annexin A2 and S100A10 or Ncan coexpression with Apolipoprotein H, APOH (coding for B2-glycoprotein 1), Tyrosinase (TYR), Transmembrane protein 212 (TMEM 212), and Fibrous sheath-interacting protein 2 (FSIP2) might hold considerable importance in the context of astrocyte response and brain repair.

Annexin A2/S100A10 complex, known to modulate crucial cellular functions like proliferation, repair, and apoptosis, underlines the intricate relationship between these proteins (as discussed in a recent review^99^). Notably, the interdependence of Annexin A2 and S100A10, with S100A10 relying on Annexin A2 for intracellular survival and the latter requiring S100A10 to enhance its affinity and participation in calcium-dependent processes, underscores their collaborative impact. Investigating whether Acan and Bcan expression profiles influence the Annexin A2/S100A10 complex at the core and peripheral regions of the injury may hold prognostic and therapeutic value, warranting further validation. Different studies have reported CSPG binding to members of the annexin family,^100^ S100A10 upregulation in A2 neuroprotective astrocytes,^42,101^ and Annexin A2 dimerization by S100A10 in the SCI model, which played a pivotal role in reactive astrocyte proliferation.^43^

In parallel, we did not identify a direct biological connection between Ncan–APOH and TYR or Ncan–TMEM 212 and FSIP2 in the context of brain injury. However, a recent study reported detectable β2-GPI (APOH) expression in the brain parenchyma and endothelium using a stroke model (Middle Cerebral Artery Occlusion).^102^ The research findings indicated that brain hypoxia and blood flow restriction lead to the upregulation of endothelial β2-GPI, which promotes neurovascular inflammation and neuron phagocytosis. It suggests that β2-GPI may be a new mediator of brain lesions following ischemic stroke;^102^ moreover, another study reported Serum β2-GPI increased levels in ischemic stroke patients that were associated with severe stroke scores and clinical outcomes.

Next, we analyzed astrocyte morphologies based on GFAP staining. In the early twentieth century, anatomists correlated astrocyte morphology with their roles in the brain and neuropathologies.^103^ Under neurological diseases, astrocyte reactivity involves hyperplasia, cell proliferation, and upregulation of the cytoplasmic proteins, GFAP, vimentin, and S100β.^104^ Subsequently, this induces a complex branch arborization and hypertrophy of the astrocyte cell soma, later identified as a reactive state.^105−107^ While we focused on GFAP, recognizing its significance as an intermediate filament associated with astrogliosis and a key component of the cytoskeleton, alternative approaches to stain the entire process to explore astrocyte cytoarchitecture may elucidate more details about the morphology.

Our PA-gel-based microenvironment platforms could support the presence of stellate, bipolar, and even perivascular cells. These morphologies, typically challenging to observe in 2D cultures and reported only in 3D models,^59,61^ were successfully maintained for at least a week (7-dps) in our 2D PA hydrogel models. While 3D models may offer additional insights into cellular behavior in a more physiological context, we chose to maintain our focus on the 2D platform to ensure experimental consistency and interpretation of the results. The presence of typical morphologies usually observed more in 3D models and *in vivo* environments underscores the considerable potential of our platforms to preserve *in vivo* morphologies, including the rarest ones, enhancing the reliability of data acquisition.

While the stiff PA-gel group had the highest number of cells with typical *in vitro* morphologies, they comprised less than 50% of the total. Rigid substrates commonly lead to maximal spreading and flatness, involving cytoskeleton reorganization and focal adhesion site adjustments.^108^ Therefore, hydrogel’s rigidity might have facilitated astrocyte branch spreading, contributing to the observed morphologies.

Our morphological analysis also revealed that astrocytes cultured on the stiff PA-gel group (1 kPa) exhibited a circular and less complex shape compared with those on the soft (300 Pa) and the intermediate-PA-gel group (800 Pa). In contrast, astrocytes cultured on the soft PA-gel had greater area and perimeter and presented more branches than those from the stiff PA-gel group, indicating the enrichment of primary and secondary branches. In the literature, a significant number of branches and the presence of secondary branches are typically associated with astrocytic response during the process of astrogliosis *in vivo*.^109^ These findings collectively suggest that substrate softness significantly influences astrocyte morphology, increasing branching and complexity.

## Conclusions

The data obtained from the 2D *in vitro* models using PA-gels showed that the soft PA-gel maintained astrocytes in a reactive state, while the stiff PA-gel induced a physiological phenotype similar to nonreactive, basal astrocytes. Furthermore, the intermediate PA-gel significantly influenced astrocytes expressing the proteoglycans aggrecan and neurocan and allowed for the occurrence of basal astrocyte morphologies. We highlight the significance of matrix rigidity in modulating astrocyte reactivity and emphasize the importance of designing substrates that replicate the natural microenvironment to comprehend the role of mechanobiology in astrogliosis. Results advance the understanding of astrocyte mechanotransduction processes within the injury region and propose PA-gel-based models for studying the reactive astrocyte response to brain damage.

## Supporting Information Available

Astrocyte immunocharacterization and reactive response on PA-gel-based microenvironment platforms and glass (control group) 3-dps; astrocyte immunocharacterization and reactive response on PA-gels-based microenvironment platforms and glass (control group) 7-dps; representative images of Acan^+^, Ncan^+^, and Bcan^+^ astrocytes after 3 and 7 days cultured on gels of 300 Pa, 800 Pa, and 1 kPa, and glass, used as control; Brevican isoforms accordingly to the astrocyte response on PA-gels-based injury microenvironment; Bcan immunolabeling of controlled cortical impact injury; representative images depicting astrocyte morphologies (PDF)

## ABBREVIATIONS

ACAN: Aggrecan
AFM: Atomic Force Microscopy
BCAN: Brevican
CCI: Controlled Cortical Impact
CSPG: Chondroitin Sulfate Proteoglycan
ECM: Extracellular Matrix
IFN γ: Interferon γ
IL β: Interleukin β
IL 6: Interleukin 6
NCAN: Neurocan
NND: Nearest Neighbor Distance
PA-gel: Polyacrylamide Gel
Pa: Pascal
S100β: Calcium-Binding Protein β
SCI: Spinal Cord Injury
SNT: Simple Neurite Tracer
TBI: Traumatic Brain Injury
