# Supplementary material for: Mechanobiological Modulation of In Vitro Astrocyte Reactivity Using Variable Gel Stiffness
Source: ACS Biomater Sci Eng. 2024 Jun 13;10(7):4279–96. doi: 10.1021/acsbiomaterials.4c00229 (PMC11234334; doi:10.1021/acsbiomaterials.4c00229)
Supplement: Supplementary file 1 — ab4c00229_si_001.pdf [file ab4c00229_si_001.pdf]

Mechanobiological modulation of *in vitro* astrocyte reactivity using variable gel stiffness

AUTHOR NAMES: *Julia C. Benincasa[a], Marianne I. Madias[b], Rebecca M. Kandell[b], Lina M. Delgado-Garcia[a], Adam J. Engler[b], Ester J. Kwon[b], Marimelia A. Porcionatto*[*, a]

AUTHOR ADDRESS:

[a] *Department of Biochemistry, Escola Paulista de Medicina, Universidade Federal de São Paulo, São Paulo 04039032, Brazil.*

[b] *Department of Bioengineering, University of California San Diego, La Jolla, California 92093, United States.*

SUPPORTING INFORMATION

[*] Corresponding author: marimelia.porcionatto@unifesp.br

Phone: +55(11)55764969

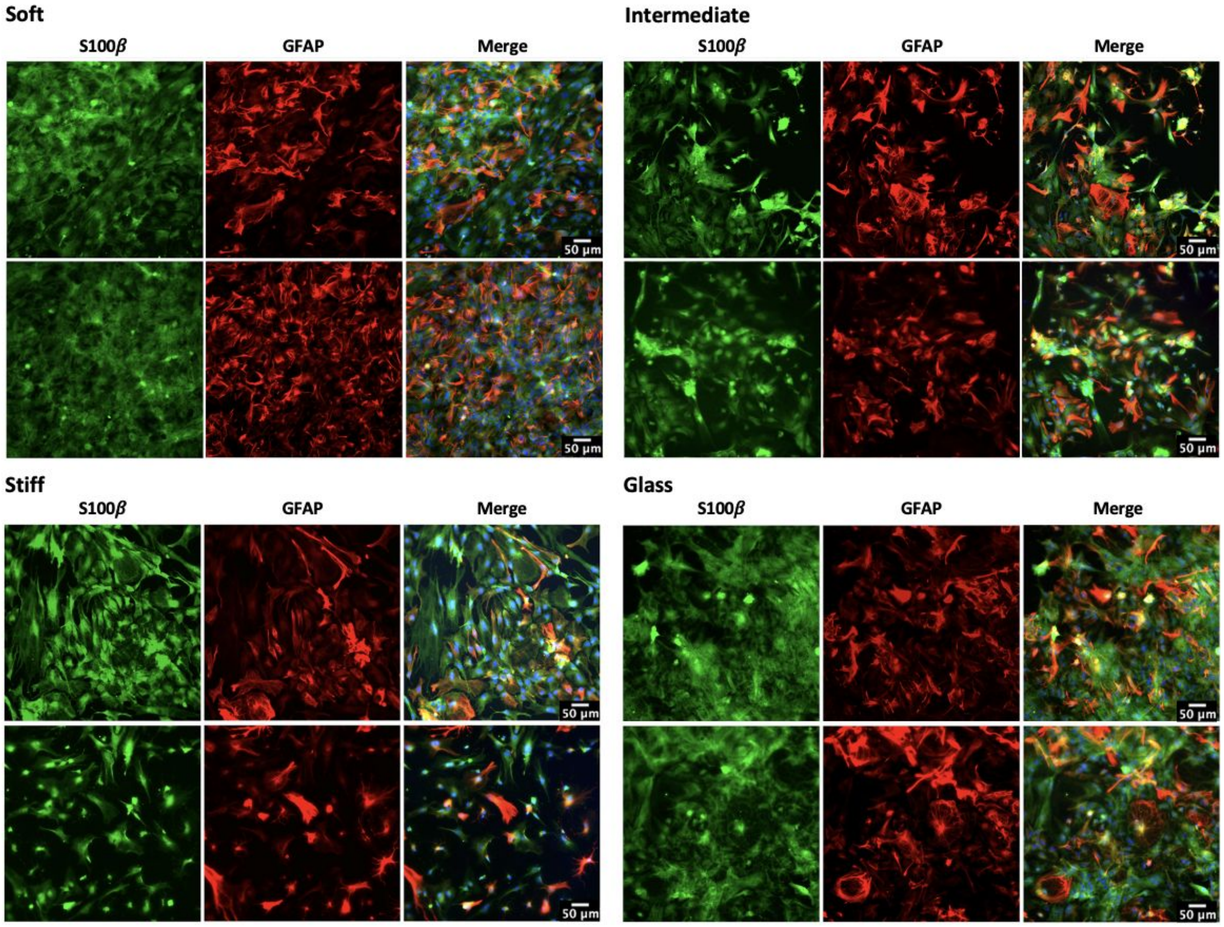

Supplementary Figure 1. Astrocyte immunocharacterization and reactive response on PA-gels-based microenvironment platforms and glass (control group) 3-dps. Representative images of GFAP and S100β immunolabeling. Scale bar 50 µm.

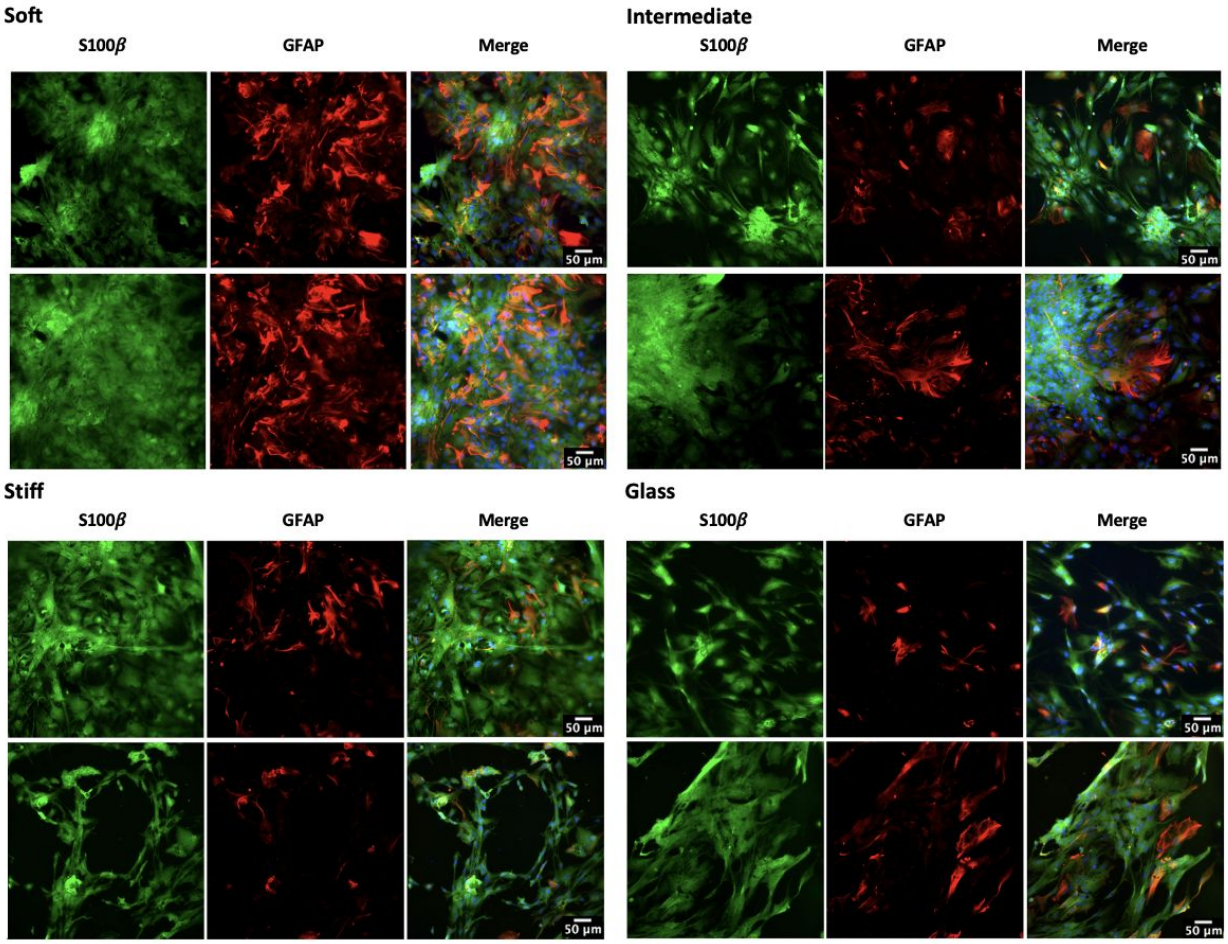

Supplementary Figure 2. Astrocyte immunocharacterization and reactive response on PA-gels-based microenvironment platforms and glass (control group) 7-dps. Representative images of GFAP and S100β immunolabeling. Scale bar 50 μm.

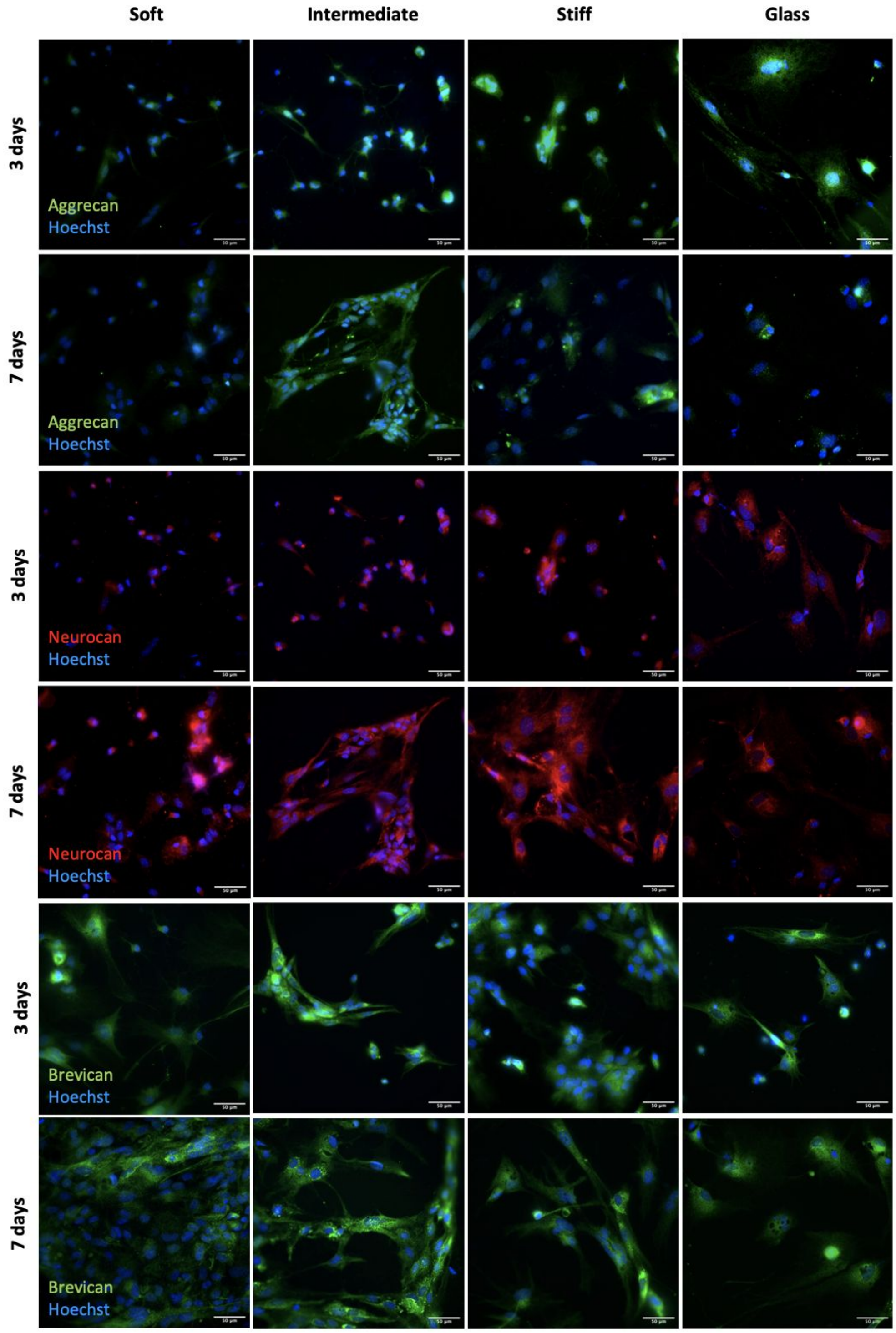

Supplementary Figure 3. Representative images of Acan$^+$, Ncan$^+$, and Bcan$^+$ astrocytes after three and seven days cultured on gels of 300 Pa, 800 Pa, and 1 kPa, and glass was used as control. Scale 50 μm.

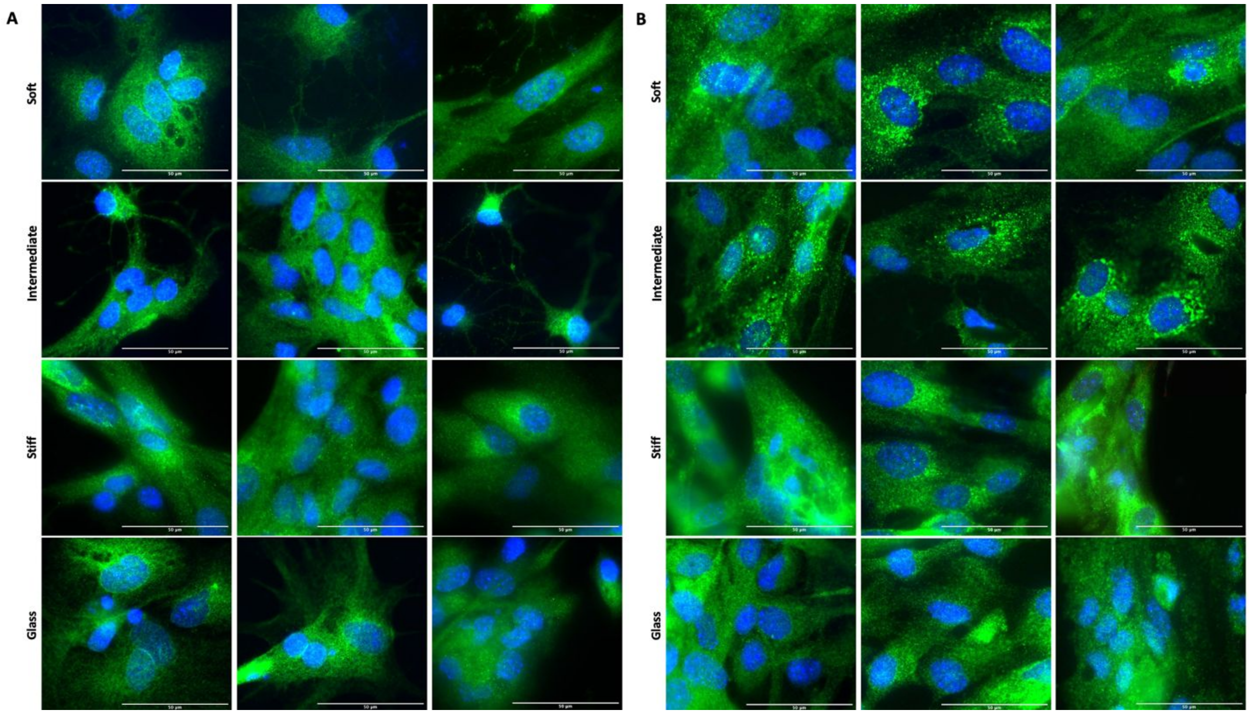

Supplementary Figure 4. Brevican isoforms according to the astrocyte response on PA-gels-based injury microenvironment. A) Representative images of Bcan^{+} astrocytes on soft-, intermediate, stiff- PA-gel and control groups, 3-dps. B) Representative images of Bcan^{+} astrocytes on soft-, intermediate-, stiff PA-gel and control groups, 7-dps. Suggestive presence of soluble Bcan isoform on soft- and intermediate PA-gel, 7-dps. Scale bar 50 µm.

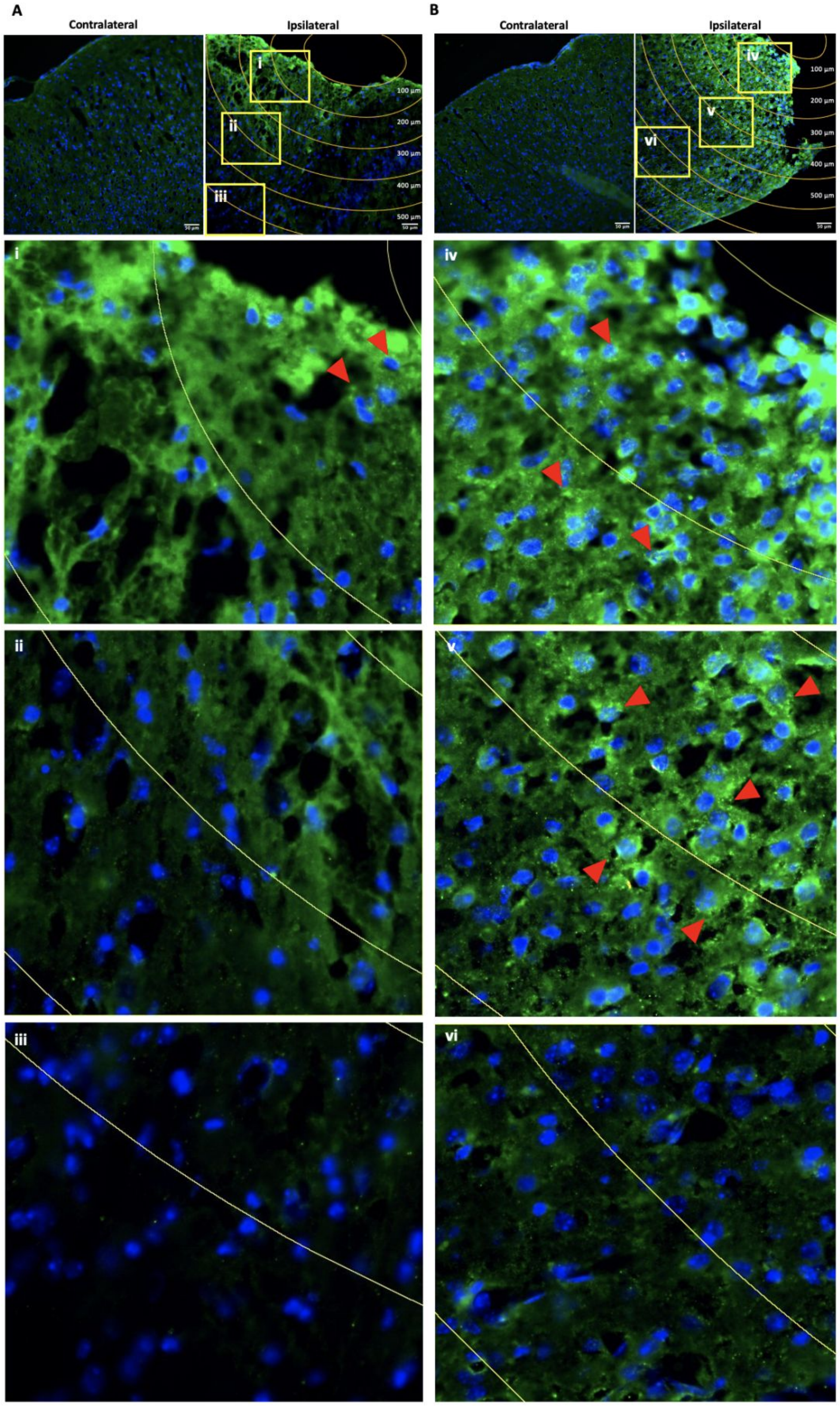

Supplementary Figure 5. Bcan immunolabeling of controlled cortical impact injury. A) Representative images of brain tissues showing contralateral and ipsilateral hemispheres 3-days

post TBI model (n=3). B) Representative images of brain tissues showing contralateral and ipsilateral hemispheres 7-days post TBI model (n=3). Detailed views showcase regions ranging from 100 to 600 μm from the injury, revealing vesicle-like structures (red arrows) predominantly observed in areas proximal to the injury site, as highlighted in i (100-200 μm from injury), iv (100-200 μm from injury), and v (300-400 μm). Scale bar: 50 μm

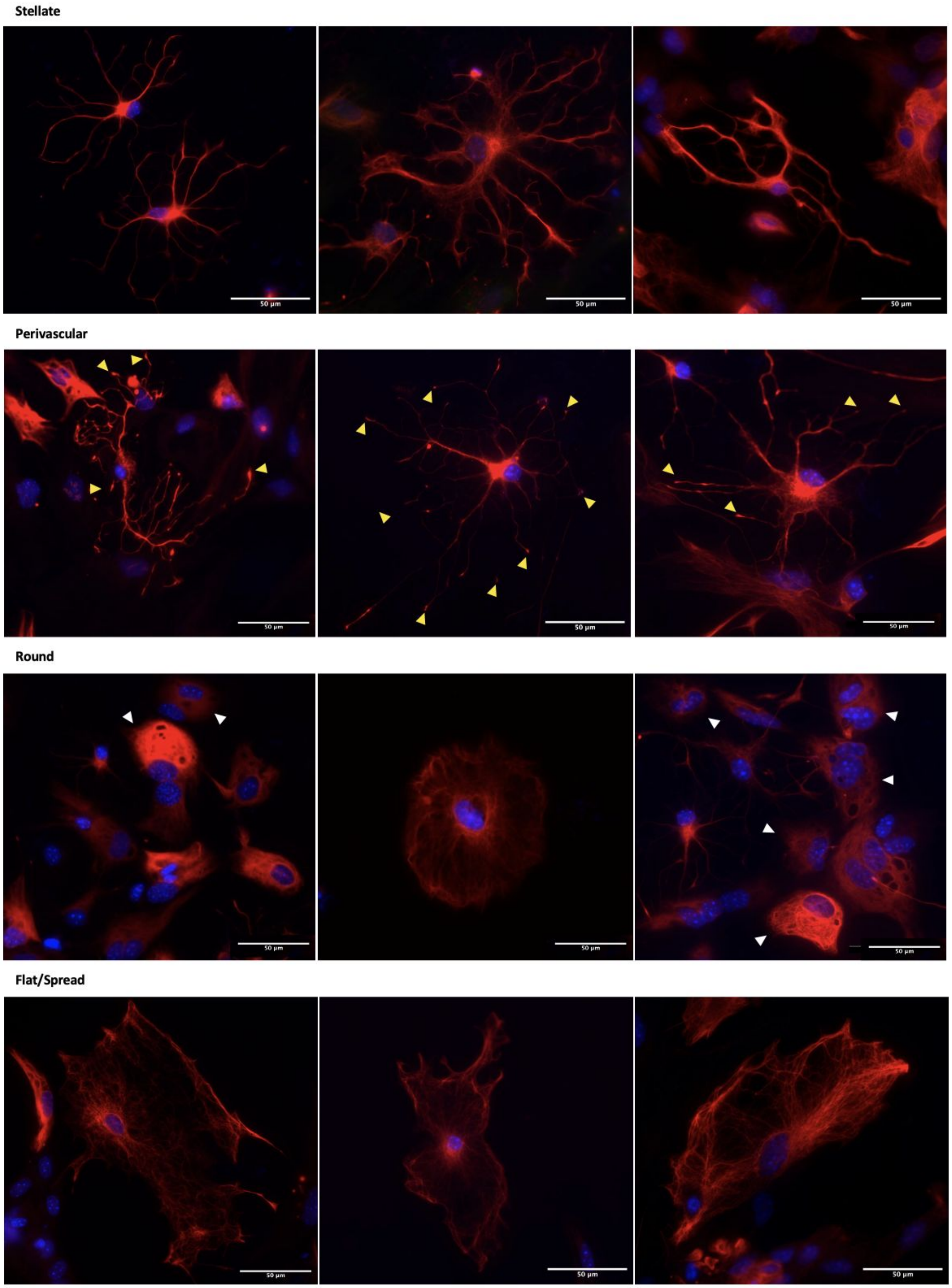

Supplementary Figure 6. Representative images depicting astrocyte morphologies: Stellate, characterized by long branches extending from a compact cell body; Perivascular (yellow arrows indicate end-feet at the extremities); Round, exhibiting a lack of branching from the cell body

(white arrows denote all rounded cells); and Flat/Spread, the predominant morphology observed in in vitro models, featuring polygonal shape and short branches emerging from the cell body.
